# Cognitive load-dependent effects of HD-tDCS on the executive vigilance decrement: insights from aperiodic EEG activity

**DOI:** 10.3389/fcogn.2025.1677285

**Published:** 2026-01-22

**Authors:** Klara Hemmerich, Juan Lupiáñez, Elisa Martín-Arévalo, Roi Cohen Kadosh

**Affiliations:** 1 Department of Psychology and Cognitive Science, University of Trento, Rovereto, Italy; 2 Department of Experimental Psychology and Mind, Brain, and Behavior Research Center (CIMCYC), Universidad de Granada, Granada, Spain; 3 School of Psychology, University of Surrey, Guildford, United Kingdom

**Keywords:** aperiodic exponent, aperiodic offset, EEG, executive vigilance, HD-tDCS, spectral parameterization, vigilance

## Abstract

**Introduction:**

This study investigated cognitive-load-dependent effects of high-definition transcranial direct current stimulation (HD-tDCS) on the executive vigilance (EV) decrement and its modulation by aperiodic electroencephalography (EEG) markers. Given the relevance of vigilance for daily functioning and its susceptibility to decline over time, we examined whether HD-tDCS could counteract this decrement under varying cognitive demands.

**Methods:**

In a between-participant design (*N* = 180), anodal HD-tDCS was applied over the right posterior parietal cortex (rPPC) during performance of a single-, dual-, or triple-task, with on-task EEG recorded pre- and post-stimulation. Power spectra were parametrized to extract aperiodic (non-oscillatory) components; the aperiodic exponent and offset across two frequency ranges (1–35 and 30–45 Hz).

**Results:**

HD-tDCS induced a reduction in the aperiodic exponent within the 30–45 Hz range (i.e., flattening of the spectral slope), consistent with increased cortical excitation. This change was associated with a mitigated EV decrement under high task demand and an exacerbated decrement under low demand, suggesting a mechanistic link between changes in excitation/inhibition balance and behavioral outcomes. However, these effects reached significance only under a directional hypothesis and seemed to be obscured by a push-pull relationship with the aperiodic offset, indicating a more complex interaction between local excitability and broadband spectral dynamics. Baseline aperiodic markers did not significantly moderate the stimulation effect but predicted overall task performance, independent of stimulation.

**Discussion:**

These findings suggest a mechanistic understanding of how endogenous neural activity, specifically, aperiodic EEG features, modulates brain stimulation outcomes. By demonstrating that HD-tDCS effects vary as a function of cognitive load and spectral dynamics, the study underscores the need for future research, centered on refined, state-sensitive stimulation protocols to mitigate the EV decrement.

## 1 Introduction

Transcranial direct current stimulation (tDCS) has the potential to modulate cognitive performance by altering cortical excitability (Antal et al., 2022; Kuo and Nitsche, 2012). Given this mechanism, a growing body of research has tested tDCS as a tool to modulate attentional functioning (Roy et al., 2015), and particularly vigilance (Brosnan et al., 2018; Coulborn et al., 2020; Hemmerich et al., 2023, 2024; Luna et al., 2020; Martínez-Pérez et al., 2023; Nelson et al., 2014). Vigilance—understood as the ability to detect infrequent but critical stimuli (Mackworth, 1948)—degrades as time-on-task progresses, a phenomenon known as vigilance decrement (Warm et al., 2008; Hemmerich et al., 2025). Essential for numerous daily activities such as driving (Wundersitz, 2019), vigilance is also crucial in work environments that require supervision or detection of threats (Barger et al., 2006; Kharoufah et al., 2018; Krüger and Suchan, 2015). Additionally, vigilance is susceptible to deterioration due to atypical brain development (Pievsky and McGrath, 2018) and acquired brain injury (Catroppa and Anderson, 2005). The potential negative consequences of the vigilance decrement in these different scenarios motivate the in-depth study of tDCS protocols that may help to understand and mitigate it. Importantly, this examination should extend beyond the behavioral effect of brain stimulation and investigate additional markers, such as electroencephalography (EEG). EEG data could provide insights into: (i) neurophysiological markers of task performance and its degradation with time-on-task, (ii) tDCS-dependent changes in neurophysiology that can explain the mechanisms of the effects of tDCS on behavior, and (iii) potential baseline neurophysiological predictors of tDCS outcomes.

Regarding applications of tDCS to mitigate the vigilance decrement, recent studies have shown that anodal high-definition tDCS (HD-tDCS) over the right posterior parietal cortex (rPPC) effectively mitigated the decrement of executive vigilance (Hemmerich et al., 2023; Luna et al., 2020); wherein executive vigilance (EV) was understood as the ability to monitor the environment and detect rare but critical stimuli, requiring deliberation in determining whether a critical stimulus is present (i.e., not completely automatic responses; Coll-Martín et al., 2023; Hemmerich et al., 2025; Luna et al., 2018). Further research showed that the mitigatory effects of HD-tDCS on the EV decrement were dependent on cognitive load (Hemmerich et al., 2024). Under low demand conditions (i.e., single and dual task), the EV decrement was relatively small and HD-tDCS did not modulate performance; if anything, the single task condition showed a non-significant trend toward a slight detrimental effect of HD-tDCS. On the contrary, under a condition of high—but not overtaxing—demand (i.e., triple task), the EV decrement was more pronounced, but mitigated by HD-tDCS (Hemmerich et al., 2024). In addition, other research has reported detrimental effects of conventional anodal tDCS over the rPPC under overtaxing cognitive load conditions (Roe et al., 2016). These results highlight the intricacies of functional targeting in tDCS, which entails not only selecting an anatomically relevant stimulation target, but also appropriately activating relevant neural connections in the target region via an external task to further enhance tDCS outcomes (Bikson et al., 2013; Miniussi et al., 2013). In this sense, brain states of more extreme under- or over-demand are likely not to interact in a beneficial manner with the external administration of tDCS. Concurrent with this idea, anodal tDCS can have different effects during the process of learning a new task. In the initial stages, where excess neural noise may be prevalent (potentially due to overload due to not having automated the task), further enhancement of cortical excitability via tDCS showed no behavioral benefit (Dockery et al., 2009). However, once the task was well-learned, anodal tDCS led to enhanced performance (Dockery et al., 2009). The use of EEG measures offers, in combination with the application of tDCS, the exciting opportunity to further validate and understand how tDCS may interact with ongoing brain states, where indirect measures of cortical excitability could be of particular interest to investigate (given that it may link task-related effects and mechanistic effects of tDCS).

**Figure 1:**
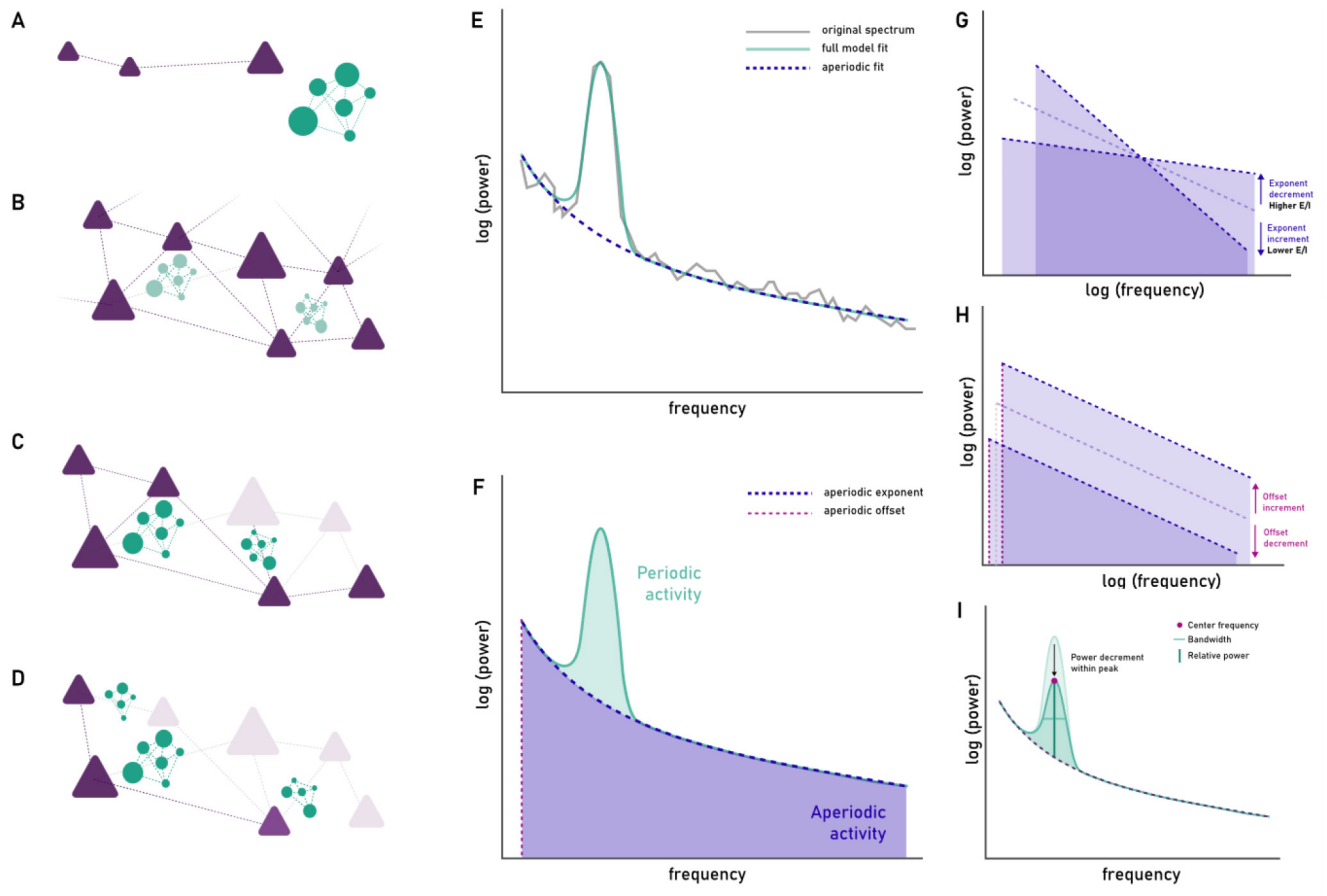
**(A)** Excitatory pyramidal neurons (left, purple triangles) and clusters of inhibitory neurons (right, green circles). **(B)** With adequate E/I balance, in response to an input, a path is shaped by inhibition, to excite relevant connections. **(C)** An example of runaway excitation, where a lack of inhibition leads to an excessive and indiscriminate excitation of other excitatory cells. **(D)** An example of an excess of inhibition that would lead the excitatory input to die out before reaching the appropriate cells to emit a response. **(E)** To parametrize the power spectrum, a model (green) is fitted to the real data (gray), obtaining the aperiodic exponent (dark blue dashed line) that is fitted to the modeled data. **(F)** The parametrized power spectrum allows disentangling aperiodic activity (the exponent, *x*, in 1/*f*^*x*^, and the offset, *y*-intercept) from periodic activity (peaks identified above the aperiodic exponent, centered around a certain frequency). **(G)** The aperiodic exponent can reflect shifts in the relative power at lower frequencies to power at higher frequencies: an increment of the exponent reflects a steeper spectral slope, and thus a lower E/I balance, whereas a decrement of the exponent, indicates a flatter spectral slope, and thus an increment of the E/I balance. **(H)** The aperiodic offset can reflect broadband shifts of power across the complete frequency spectrum that is being analyzed, which might reflect spiking across larger populations of neurons. **(I)** The periodic data comprises the peaks detected above the aperiodic exponent, with each peak identified by its center frequency, bandwidth, and power.

Cortical excitability depends on a delicately balanced homeostatic state—referred to as criticality or self-organized criticality—where, in response to an input, an adequate level of excitatory and inhibitory (i.e., E/I balance) connections shapes the correct path to emit an output (Ahmad et al., 2022; Krause et al., 2013). At the cellular level, this involves activity from excitatory pyramidal cells and clusters of inhibitory neurons (Figure 1A). A balanced state supports efficient signal propagation (Figure 1B). By contrast, imbalances can disrupt neural communication: excessive excitation, due to insufficient inhibitory control, can lead to a disorganized neural state or, in extreme cases, epileptic seizures (Figure 1C; Žiburkus et al., 2013), whereas excessive inhibition prevents signals from propagating along their intended pathways, thereby dampening neural responses (Figure 1D; Poil et al., 2012). The effect of tDCS on cognition could be explained by how the technique shifts this E/I balance (Krause et al., 2013).

While the E/I balance may be inferred more directly from intracranial recordings, aperiodic parameters extracted from EEG recordings may offer a valuable non-invasive approach to exploring global activity across neuronal populations to infer this global cortical state (Ahmad et al., 2022; Gao et al., 2017; Weber et al., 2020). EEG power spectra typically follow a 1/*f*-like distribution, where lower frequencies have higher power and power diminishes as frequency increases (Figure 1E). This characteristic pattern reflects scale-free neural activity and can be described using the function 1/*f*^*x*^, where *f* denotes frequency and *x* represents the aperiodic exponent, a parameter indicating how steeply power decreases with increasing frequency, whereas the *y*-intercept of this slope, the aperiodic offset, captures broadband shifts in power across all frequencies (see Figure 1F, Donoghue et al., 2020b, 2021). While already previously explored (Gilden, 2001), a recent resurgence of interest in aperiodic EEG data has facilitated the emergence of new methods of extraction of these measures (Donoghue et al., 2020a,b; Gerster et al., 2022). Spectral parametrization (Donoghue et al., 2020b) fits a model (green line in Figure 1E) over the original power density spectrum (gray line in Figure 1E), adjusting to the existing peaks, which then allows fitting the aperiodic exponent (blue dashed line in Figure 1E) to the modeled data, disentangling aperiodic activity (blue surface in Figure 1F)—not contained by any predominant temporal scale—from periodic activity (green surface in Figure 1F)—constrained to oscillatory activity in a specific narrowband frequency (He, 2014). This approach facilitates the independent analysis of aperiodic metrics, devoid of the influence of periodic activity (Donoghue et al., 2020a,b), such as changes in the aperiodic exponent, reflecting a steepening or flattening of the spectral slope (Figure 1G), or the aperiodic offset, reflecting a broadband shift up- or down-wards of the whole power spectrum (Figure 1H). Additionally, this approach also allows inspecting periodic activity (i.e., peaks above the aperiodic exponent) in a certain frequency band/range, devoid of the background aperiodic activity (Figure 1I). Importantly, recent animal research has shown that the EEG spectral slope reflects the relative activity of excitatory pyramidal neurons and inhibitory interneuron activity, but only during low excitatory states (Lendner et al., 2023), highlighting that the link between cellular and EEG-based E/I balance is context-dependent.

While the aperiodic exponent and offset might offer relevant insights into vigilance functioning, this link has not been explored yet, as current associations to vigilance have been made more prominently with periodic EEG data (without separating it from aperiodic contributions). Specifically, alpha power has shown to increase with time-on-task (Boksem et al., 2005; Craig et al., 2012; Hemmerich et al., 2023; Luna et al., 2020), potentially reflecting attenuated information processing (Pershin et al., 2023), or an increased need to inhibit task-irrelevant stimuli (Clayton et al., 2015). However, recent evidence supports the idea that the aperiodic exponent can function as a proxy of the E/I balance, through computational simulations, intracranial recordings, and external manipulations known to alter the ratio of excitation to inhibition. General anesthesia administration has been associated with steeper power spectra, indicating a reduced E/I balance, in both animal intracranial (Gao et al., 2017; Lee et al., 2020; Medel et al., 2023) and human recordings (Lendner et al., 2020). Instead, a flattening of power spectra, indicating an increased E/I balance, has been observed when transitioning from sleep to wakefulness (Lendner et al., 2020), and from rest to performing a task (button-press or visuomotor: He et al., 2010; visuomotor: Podvalny et al., 2015). Modality switching during a detection task was associated with a flattening of spectral slopes in regions related to the attended modality (Waschke et al., 2021). However, Pathania et al. (2021) observed steeper power spectra (i.e., lower E/I balance) during a videogame task compared to a rest period. Regarding cognitive control, several studies observe flatter power spectra when inhibition is deployed (e.g., NoGo trials of a Go-NoGo task or trials presenting an incongruency), than those where it is not needed (e.g., Go trials or congruent trials; Pertermann et al., 2019; Pi et al., 2024; Zhang et al., 2023). In line with this, Ouyang et al. (2020) found that faster processing speed in an object recognition task was predicted by steeper slopes in resting-state EEG (rs-EEG) data recorded prior to the task, potentially reflecting individual capacity to better inhibit task-irrelevant information to permit more efficient information processing. These findings point to a more linear relationship between induced inhibition and excitation in simpler contexts, whereas in more complex task contexts, especially those requiring inhibition of task-irrelevant information, this linearity may disappear. In addition to the aperiodic exponent, the offset may also offer insight into cortical dynamics relevant to cognition. The aperiodic offset has been associated with neuronal spiking in scalp EEG data (Manning et al., 2009). Manning et al. (2009) suggest that some effects, previously attributed to the gamma band, might actually reflect broadband power increases. The aperiodic offset increases during early brain maturation (Rico-Picó et al., 2023), but decreases from child- to adulthood (McKeon et al., 2024), and with older age (Donoghue et al., 2020b). It has also been related to performance in a task-dependent manner: a higher offset predicted better marksmanship performance early in training but worse performance later on (Immink et al., 2021), and was associated with improved working memory but reduced attention and processing speed following exercise (Braunsmann et al., 2024). Similarly, Ouyang et al. (2020) observed that a higher offset at rest was associated with slower processing speed. Compared to the exponent, the offset has received less attention in recent literature, and its functional significance remains less well-defined. Still, current evidence suggests it may represent a distinct, state-sensitive neural marker influenced by task demands and cognitive load.

While the aperiodic exponent and offset could offer valuable insights into vigilance functioning and the mechanisms underlying the effectiveness of tDCS in mitigating the vigilance decrement, neither of these links has been previously explored. More broadly still, emerging evidence suggests that different tES techniques can both modulate and be affected by aperiodic parameters. For example, van Bueren et al. (2023) found that active tRNS reduced the aperiodic exponent (i.e., flattened the spectral slope) in rs-EEG compared to sham, and steeper pre-stimulation aperiodic slopes predicted greater learning gains during an arithmetic learning task. Using a similar task, Sheffield et al. (2020) reported enhanced tRNS efficacy in participants with steeper slopes. In ADHD, children typically exhibit higher exponents (Dakwar-Kawar et al., 2024; Robertson et al., 2019), and a combined tRNS and cognitive training intervention led to flatter slopes post-intervention (Dakwar-Kawar et al., 2023), along with improved parent-rated symptom scores, though a causal link between both measures was not directly tested. Studies using transcranial alternating current stimulation (tACS) and tDCS show mixed effects: gamma-range (40 Hz) tACS reduced the offset (Masina et al., 2025); no changes were found after tACS targeting individual alpha frequency (Kasten et al., 2024) nor the theta and gamma bands (Venugopal et al., 2025). In patients with drug-resistant epilepsy, tDCS increased both aperiodic exponent and offset, while alpha-tACS showed a delayed reduction of the exponent (Simula et al., 2024). Gao et al. (2025) reported an increased exponent after tDCS, but only when combined with methylphenidate (a stimulant).

Complementing EEG approaches, magnetic resonance spectroscopy (MRS) provides a molecular-level proxy of E/I balance by quantifying concentrations of excitatory glutamate and inhibitory GABA in specific brain regions. The glutamate/GABA ratio is often interpreted as an index of cortical excitability (Duncan et al., 2014; Stagg et al., 2011), although the relationship is region- and context-dependent. Testing a more direct association between the two measures, McKeon et al. (2024) found that the MRS-based asymmetry between glutamate and GABA decreased in relation to EEG-based increases in the aperiodic exponent. This association was driven mostly by glutamate, as higher glutamate levels (indicative of higher excitation), were associated with a larger exponent (indicative of higher inhibition; McKeon et al., 2024). This discrepancy may be explained by the fact that MRS- and EEG-based E/I measures use different target populations, such as intra- and extra-cellular, for each method, respectively (Buzsáki et al., 2012; Dyke et al., 2017), or capture complementary aspects of E/I dynamics, where MRS reflects static, neurochemical concentrations, EEG captures dynamic, functional fluctuations in excitability (Cohen Kadosh, 2025). While MRS-based measures of E/I have been used to determine tES outcomes, using tDCS (Bunai et al., 2021; Filmer et al., 2019; Vural et al., 2024) and tRNS (van Bueren et al., 2023; Zacharopoulos et al., 2025), it should be noted that the inferences on the E/I balance are linked to the specific neuroimaging method of acquisition, highlighting the fact that interpretations relating to the E/I cannot be generalized beyond the method of acquisition.

From the current state of the literature, the following objectives for further research emerge: (i) to disentangle aperiodic and periodic components in EEG data to inspect their individual contributions and potential interactions along the tDCS-brain-behavior axis, (ii) to explore how the findings observed with other transcranial electric stimulation (tES) techniques translate to tDCS, as the effect on cortical excitability may vary among techniques (Inukai et al., 2016, albeit not consistently, see: Ho et al., 2015); and (iii) to investigate the potential causal link between tDCS induced changes in electrophysiology and tDCS induced behavioral changes. This could lead to a better understanding of tES mechanisms and improve the ability to infer causality in tES interventions (Bergmann and Hartwigsen, 2021; Harty et al., 2017).

### 1.1 The present study

The present study aimed to explore whether aperiodic and periodic EEG markers could predict or explain the efficacy of HD-tDCS in mitigating the EV decrement. The rationale and design of this study were pre-registered on the Open Science Framework (OSF, osf.io/umjc8), though the study was conceived after data collection for prior research, some deviations from the preregistration occurred during analyses. We here detail the pre-registered hypotheses and highlight the changes and additional hypotheses that were introduced. Specifically, we pre-registered the hypothesis that HD-tDCS would increase cortical excitation (indexed by a reduction in the aperiodic exponent), and initially predicted polarity-dependent electrode-specific effects. However, analyses revealed a general effect across electrodes, prompting the use of averaged values for remaining analyses. Based on this, additional hypotheses were introduced: we expected that the increased excitation resulting from the application of HD-tDCS would explain the beneficial effects of HD-tDCS on the EV decrement observed in the triple task; and potentially the marginal detrimental effects observed in the single task (Hemmerich et al., 2023, 2024). The change in the aperiodic offset and alpha power were introduced as additional mediators that could potentially explain HD-tDCS outcomes. A second preregistered aim was to test whether the baseline aperiodic exponent could predict HD-tDCS efficacy, in line with prior tRNS studies (Sheffield et al., 2020; van Bueren et al., 2023). We also explored whether baseline offset and alpha power might moderate outcomes. While some hypotheses were adapted based on preliminary results, the central goal remained to clarify the neurophysiological mechanisms underlying HD-tDCS effects on the vigilance decrement. Together, while acknowledging the exploratory adaptations made to the pre-registered plan (as later hypotheses and analysis steps were contingent on earlier findings and thus adjusted in response to the data), the main aim remained to investigate the neurophysiological mechanisms underlying tDCS effects on the vigilance decrement as a function of task-induced brain states.

## 2 Materials and methods

### 2.1 Participants

The total sample was comprised of 180 participants. This data is part of a larger study exploring whether the EV decrement can be mitigated by HD-tDCS applied over the rPPC, and its dependence on cognitive load (Hemmerich et al., 2023, 2024). The combination of these two between-participant experimental conditions (Stimulation Condition × Cognitive Load Condition) led to 30 participants per experimental condition. Given that this data was collected as part of prior experiments, sample size calculations are provided in previous reports of this data (see Hemmerich et al., 2023). Participants were selected according to the following inclusion criteria: aged between 18 and 35 years, right-handed, with normal or corrected-to-normal vision, no known neurological or psychiatric conditions, and no safety contraindications for receiving tES (Rossi et al., 2009; Rossini et al., 2015) or undergoing magnetic resonance imaging (MRI). All participants gave signed consent and received 10€/h in exchange for their participation. This study was embedded in larger research projects (PID2020-114790GB-I00 and B-CTS-132-UGR20) approved by the Ethical Committee of the University of Granada (2442/CEIH/2021 and 1188/CEIH/2020), following ethical standards of the 1964 Declaration of Helsinki (last update: Helsinki, 2024).

### 2.2 Behavioral measures

**Figure 2:**
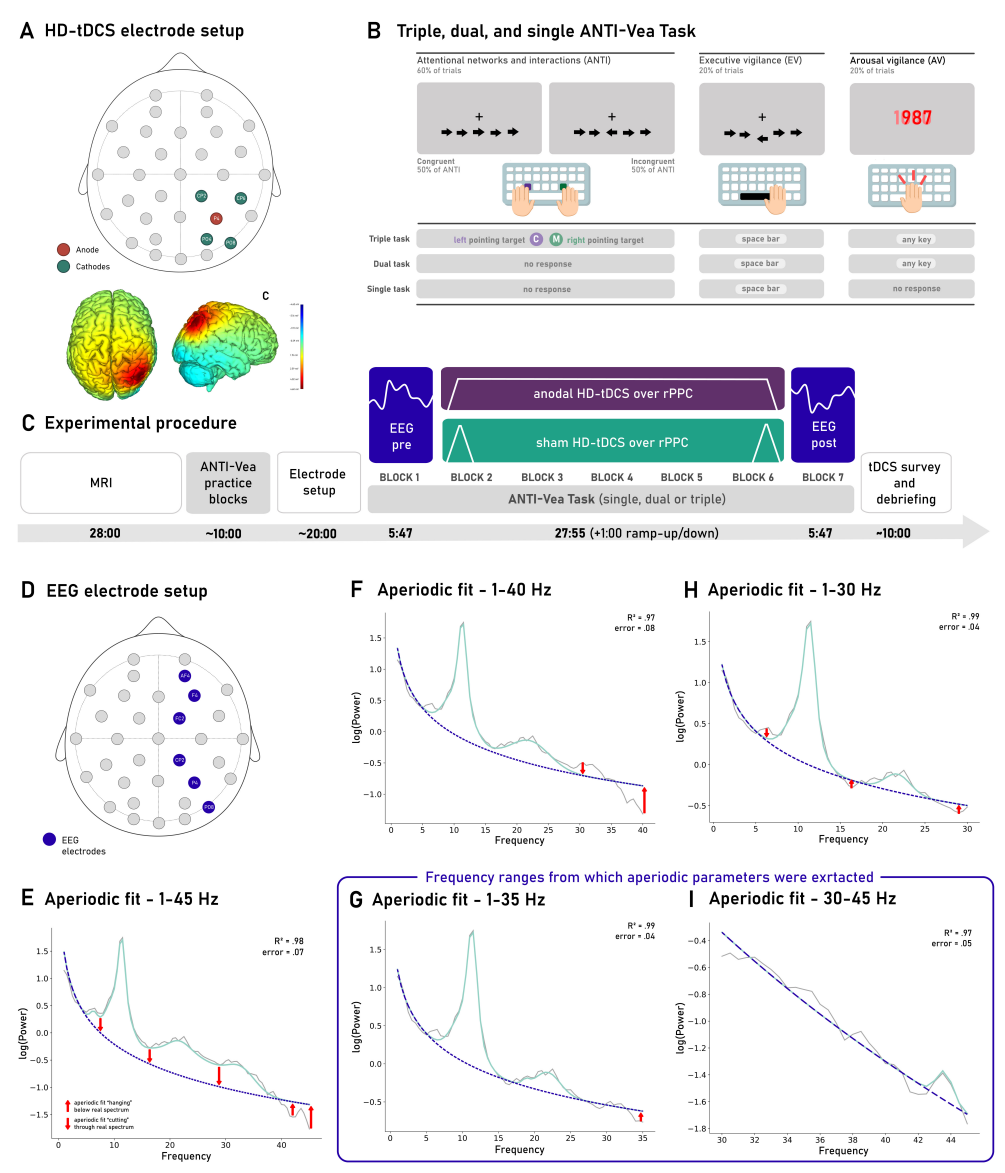
**(A)** Electrode setup for HD-tDCS procedure targeting the rPPC (anode in red and cathodes in green), and below, simulated e-field resulting from the HD-tDCS protocol, top and right lateral view. **(B)** ANTI-Vea task procedure for ANTI, EV, and AV trials, and configuration of triple, dual, and single task versions. **(C)** Procedure of the experimental session. **(D)** Electrode setup for EEG recording (dark blue electrodes) from which aperiodic parameters were extracted for each participant, each electrode, each pre-post HD-tDCS on-task recording. **(E)** Aperiodic fit from one example dataset extracted from the 1 to 45 Hz frequency range, showing how, while the fit of the modeled data (light green) is adequate, the aperiodic exponent does not adequately fit to the power spectra shape (dropping below the end of peaks at intermediate frequencies, red downwards pointing arrows), and cuts of the spectrum above 35 Hz (red upwards pointing arrow). **(F–H)** Systematic reduction of the upper limit of the frequency range by 5 Hz, showing an improved fit but still a cut-off of frequencies at higher frequencies in the 1–40 Hz range, an adequate fit in the 1–35 Hz range, and a slight cut-off in the 1–30 Hz range. **(I)** Aperiodic fit in the 30–45 Hz range. The selected frequency ranges from which aperiodic parameters were extracted are highlighted within the blue box.

Participants performed either a single, dual, or triple task in order to manipulate the cognitive load evoked by the task. All three tasks were versions of the ANTI-Vea Task (Coll-Martín et al., 2023; Luna et al., 2021). The triple task corresponded to the standard ANTI-Vea task, where 60% of trials consist of a flanker task (ANTI trials), 20% of trials consist of EV trials (where the central arrow between the flankers is vertically displaced and participants need to press an alternate key upon detection), and the remaining 20% of trials are arousal vigilance (AV) trials (where a large red countdown appears in the middle of the screen and participants have to stop it as fast as possible by pressing any key). In the dual and single tasks, the same stimuli were presented, changing only the instructions and coding of correct responses. In the dual task, participants had to respond only to AV and EV trials, whereas in the single trials, only EV trials had to be responded to (for more details on these task manipulations, see Luna et al., 2022). See Figure 2B for an example of each type of trial.

### 2.3 HD-tDCS procedure

Using a single-blind procedure, HD-tDCS over the right posterior parietal cortex (rPPC) was applied online to the performance of the single, dual, or triple tasks, from the 2^nd^ to the 6^th^ task block (for ~28 min), with an intensity of either 1.5 mA (anodal stimulation group, *n* = 90) or 0 mA (sham group, *n* = 90) and a ramp-up and ramp-down of 30 s. The sham procedure consisted of ramps at the beginning to 1.5 mA and end of the stimulation period with a stimulation duration of 30 s. Stimulation was applied with a Starstim 8 device (Neuroelectrics^®^, Barcelona), using five of the total eight hybrid NG Pistim Electrodes (with a 12 mm Ag/AgCl sintered pellet, with a circular contact area of 3.14 cm^2^), set up in a 4 × 1 ring-like array with the central anode over P4, and the four surrounding cathodes over CP2, CP6, PO4, and PO8 (see Figure 2A), in order to target the rPPC (see Figure 2A for a simulation of the resulting electric field).

### 2.4 EEG procedure and pre-processing

EEG was recorded from six electrodes (AF4, F4, FC2, CP2, P4, and PO8), as depicted in Figure 2D (electrodes circled in blue), collecting on-task data before and after the application of HD-tDCS. The signal was recorded with a sampling rate of 500 Hz, a bandwidth of 0–125 Hz, and a notch filter at 50 Hz. EEG data was pre-processed with the EEGLAB toolbox v2020.0 and v2021.1 (Delorme and Makeig, 2004) run on MATLAB R2020a (The MathWorks, Inc.). A 210-s epoch was selected from the original recordings (347-s blocks) to avoid any contamination by either the ramp-up or ramp-down. Then, high-pass (0.5 Hz) and low-pass (45 Hz) filters were applied. Afterwards, Independent Component Analysis (ICA) was run to identify and reject artifacts (mainly for blinks and eye movements), and visual inspection of the data was used to reject remaining non-periodical artifacts, leaving pre-stimulation EEG datasets at an average of 209.30 s, and post-stimulation EEG datasets at 208.69 s.

For each dataset (pre- and post-stimulation EEG for each of the six EEG electrodes), power density spectra were estimated with Fast Fourier Transform (FFT) using the Welch method, with the *pwelch* function in MATLAB R2020a, with a frequency resolution of 0.5 Hz over 2-s Hann windows with a 1-s overlap. Power spectra were intentionally calculated using a lower frequency resolution, achieving sufficiently smooth spectra in order to avoid fitting peaks to noise (Gerster et al., 2022). The power density spectra were then parametrized into their aperiodic and periodic components using the “fitting oscillations and one-over-f” (FOOOF) v1.1.0 algorithm (Donoghue et al., 2020b) run in Python v3.9., with the following parameters: peak width limits [1–6], maximum number of peaks [8], minimum peak height [0.2], peak threshold [1.5] and fixed aperiodic mode. These parameters were based on the default settings, adjusted for optimal fit to the data via visual inspection of the model fit and goodness-of-fit measures (GoF): error and *R*^2^. This extraction was performed individually for each dataset, and over two different frequency ranges. It was pre-registered that a frequency range of 1–45 Hz would be used, given the interest in the gamma band (30–45 Hz) explored in a prior study (Hemmerich et al., 2023). However, while the parametrized output of the power spectra in this frequency range showed an adequate fit of the modeled data (thus, also good GoF measures), visual inspection revealed an inadequate aperiodic fit (i.e., aperiodic exponent “hanging” below the actual slope of the data, as shown in Figure 2E for one example dataset). Systematically reducing the upper frequency range limit from 45 Hz in 5 Hz steps (see Figures 2F–H), revealed an adequate fit at the 1–35 Hz frequency range [through both overall GoF measures (across electrodes: *R*^2^ = 0.98, error = 0.05), and visual inspection of the individual spectra after parametrization], which was then chosen to extract periodic and aperiodic data (**low-frequency**). To gain insight from the excluded frequency range, a second extraction of periodic and aperiodic parameters was performed over the 30–45 Hz range (**high-frequency**), which produced a good fit [through GoF measures (across electrodes: *R*^2^ = 0.97, error = 0.05) and visual inspection of the individual spectra, see Figure 2I for an example] and has been reported as providing reliable outcomes in prior research (Höhn et al., 2024; Lendner et al., 2020). GoF measures were used to filter out outliers prior to analyses, setting the threshold at 3 standard deviations (SD) error and at 2.5 SD for *R*^2^. For each dataset (still separate for each participant, recording period, and electrode) aperiodic parameters were extracted: the aperiodic exponent (slope of the exponent fit to the model that was adjusted to each power density spectrum), and the aperiodic offset (intercept at the *y*-axis of the aperiodic exponent slope). This way, the exponent and offset were obtained for each frequency range. The extraction of periodic parameters (namely, in the alpha frequency band) after spectral parametrization is detailed in Supplementary Section 1.

### 2.5 Procedure

The experimental session began with a structural MRI scan (30 min), which was collected as part of a larger research project beyond the present study's goal. For the main experiment, participants sat in a dimly lit room to complete the ANTI-Vea task with simultaneous EEG recording and HD-tDCS application. Participants started by reading task instructions (adjusted to each task version) and completing several practice blocks. After that, electrode setup for EEG and HD-tDCS was completed and the task was started. During the experimental task, pre-stimulation EEG recordings were completed during the 1st experimental block (5:47 min). Then, anodal or sham HD-tDCS over the rPPC was applied from the 2^nd^ to the 6^th^ experimental block (~28 min). During the 7^th^ and last experimental block, the post-stimulation EEG measures were recorded (5:47 min). Subjective assessments of mental and physical fatigue were completed by participants at three points: before the practice block (baseline), before (pre-task), and after the task (post-stimulation). Right after, participants completed a tES Survey, recording their subjective experience during stimulation to test the study's blinding efficacy (Fertonani et al., 2015). The experimental procedure is depicted in Figure 2C.

### 2.6 Data analysis

The EV decrement was the main measure of interest at the behavioral level, given that responses to EV trials were collected across all task load conditions, and prior findings observed a dissociated effect of rPPC HD-tDCS mitigating the EV decrement, but not the AV decrement (Hemmerich et al., 2023; Luna et al., 2020). As a more summarized effect of time-on-task effects on the EV decrement the EV Slope was obtained, as was done in prior studies (Hemmerich et al., 2023; Luna et al., 2020), by calculating the regression line for each participant's Hits (i.e., correct responses to vertically displaced targets) across Blocks 1–6 (i.e., from Baseline up to the halt of active/sham stimulation). This slope is typically negative, reflecting a decline in the % of hits across blocks of trials.

For each of the aperiodic EEG measures (exponent and offset extracted from the 1 to 35 and 30 to 45 Hz ranges) we calculated two indices: a Baseline index, filtering out data from the pre-stimulation EEG recordings, and a change-index (Δ = post-stimulation − pre-stimulation), subtracting the pre-stimulation data from the post-stimulation data at each electrode for each participant. Thus, negative values for the Δ aperiodic exponent mean that the slope became flatter (i.e., increased E/I balance), whereas positive values mean that the exponent became steeper (i.e., reduced E/I balance). On the other hand, a positive Δ aperiodic offset, would reflect an upwards shift of the power spectrum, whereas negative values, would be indicative of a downwards shift of the spectrum.

To test pre-registered hypotheses of electrode-specific effects on the aperiodic indices, each one was introduced as a dependent variable in an ANOVA, with Stimulation Condition and Task Type as between-participant factors and Electrode as a within-participant factor. For reported results from these ANOVAs, degrees of freedom are reported with Greenhouse–Geisser correction when the sphericity assumption was violated (i.e., *p* > 0.05 in Mauchly's test).

Follow-up exploratory analyses examined whether the effect of HD-tDCS on the EV decrement was mediated by pre- to post-stimulation changes in aperiodic EEG components (Aperiodic Exponent and Offset from 1 to 35 and 30 to 45 Hz range). The aperiodic components were entered as parallel mediators into a model with Stimulation Condition (sham or anodal HD-tDCS over the rPPC) as the predictor, and EV Slope as the outcome measure (Hayes' Model 4; Hayes, 2022). This approach enabled the estimation of each mediator's unique potential contribution, while accounting for potential suppressed effects (Hayes, 2022; MacKinnon et al., 2000). Parallel mediation analyses were conducted separately for each level of Task Type (single, dual, and triple), as prior behavioral analyses had revealed different direct effects of Stimulation Condition on the EV Slope (Hemmerich et al., 2024).

To test the second pre-registered hypothesis—that Baseline Aperiodic Exponent values predict HD-tDCS effects on the EV decrement—a generalized linear mixed-effects model (GLMM) was fitted using the *lme4* package (Bates et al., 2015) in RStudio, to examine the effects of EV Trial (number), Task Type, Stimulation Condition, and Baseline Aperiodic Exponent on accuracy in EV trials. The model included all main effects and their interactions: *Accuracy* ~ *EV Trial* × *Task Type* × *Stimulation Condition* × *Baseline Aperiodic Exponent (centered)*, with random slopes for trial number by participant (i.e., *EV Trial | participant*) to account for individual variability in task performance over time. A binomial family with a logit link was used, and model fitting was performed using the *bobyqa* optimizer with a maximum of 1^9^ function evaluations to ensure convergence. Analyses were run separately for the Baseline (pre-stimulation) Aperiodic Exponent averaged across electrodes for the 1–35 and the 30–45 Hz frequency ranges.

Further exploratory analyses were conducted for this research question, introducing the same data into Hayes' moderation Model 3 (Hayes, 2022). As in the prior models, the direct effect of Stimulation Condition on EV Slope was assessed, testing whether it was moderated by the baseline value of either EEG measure (i.e., pre-stimulation EEG recording), further moderated by Task Type. Therefore, these models were run separately with each baseline EEG value as a second moderator.

All mediation and moderation models were run via the PROCESS macro v.4.1.1 (Hayes, 2022) in RStudio. All continuous predictor variables were mean-centered. Percentile bootstrapping was performed using 1,000 intervals and a 95% and 90% confidence interval (CI), considering effects significant when the CI did not include zero.

**Figure 3:**
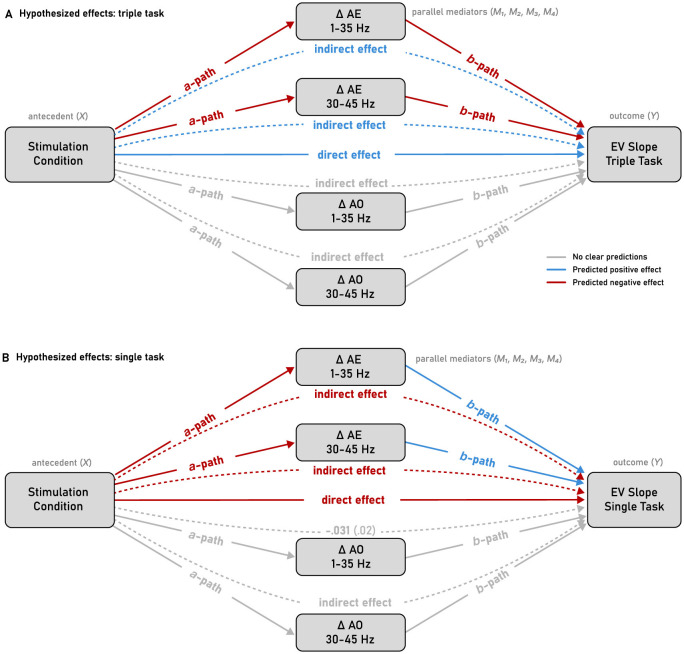
Schematic depiction of parallel mediation models and hypothesized outcomes. **(A)** Hypothesized effects to be observed in the triple-task condition: we expect an increase in excitability to be reflected in a decrement of the aperiodic exponent (negative relationship) under active anodal HD-tDCS, that, in turn, leads to a flatter executive vigilance (EV) slope (i.e., mitigated EV decrement, and thus negative relationship). Thus, the direct positive effect observed in prior research, could be explained through a positive indirect effect of the aperiodic exponent. No predictions were pre-registered or could be derived from literature for the offset. **(B)** Hypothesized effects to be observed in the single-task condition: the same effect of HD-tDCS on the aperiodic exponent as in the triple-task condition was expected (negative relationship). However, given the marginal detrimental (negative) direct effect observed in prior research, we hypothesize that this decrement of the exponent would in turn lead to a steeper EV slope (i.e., an exacerbated EV decrement, a positive relationship) and thus lead to a negative indirect effect. No predictions were pre-registered or could be derived from literature for the offset. AE, aperiodic exponent; AO, aperiodic offset.

One-sided hypothesis testing (i.e., using a wider CI of 90%) was used (i) to test hypotheses for which we had specific predictions (see Figure 3), where we expected that the same effect of the HD-tDCS protocol on the aperiodic exponent—namely a flattening of the spectral slope (i.e., a shift toward increased excitation)—could explain the beneficial effects on EV performance in the triple task group (a positive indirect effect, see Figure 3A), and the detrimental effects on EV performance in the single task group (i.e., a negative indirect effect, see Figure 3B); and (ii) for those cases where no specific hypotheses were established, to allow for more sensitive detection of potentially meaningful effects, consistent with recommendations for exploratory or early-stage mediation research (MacKinnon et al., 2010; Shrout and Bolger, 2002). Nonetheless, significant results from analyses using a 90% CI were not taken as confirmatory, but rather as preliminary findings that could suggest a potential association, which will need confirmation from future research. A common seed was used to ensure the replicability of analyses. Bootstrapping (robust resampling technique that estimates the sampling distribution of a statistic by resampling with replacement from the observed data), provides advantages in the context of these analyses as it does not rely on assumptions about the shape of the sampling distribution, which makes it particularly useful for small sample sizes or non-normally distributed data (Hayes, 2022), ensuring a more robust analysis. The *modelbt* option was activated in order to obtain bootstrapped CI's for all regression coefficients and not only the direct and indirect effects. Simple paths, direct, indirect, or moderated effects were considered significant when the CI did not include zero. Unstandardized regression coefficients (b), standard error (SE), and CI are reported in Tables in the Main Text and Supplementary material.

Finally, it was also preregistered that prior findings regarding periodic EEG results (analyzed for the triple task condition, Hemmerich et al., 2023) would be re-tested on the full sample (i.e., including the single and dual task conditions) to see whether they hold up when periodic data after spectral parametrization is used instead. Hemmerich et al. (2023) reported significant effects of HD-tDCS of power in the alpha (7.5–12.5 Hz) and gamma (30–45 Hz) range, as well as combining the two frequency bands into a joint index. These analyses could not be fully replicated in the current dataset with the parametrized periodic EEG data, as peaks in the selected frequency range were not observed for all participants (especially limited for the gamma band). Detailed results from periodic data extraction are reported in Supplementary Section 1. For peaks from the alpha band, where still a considerable part of the full sample had peaks, alpha power was introduced into mediation (for Δ Alpha Power, i.e., pre-post stimulation change) and moderation (for Baseline Alpha Power) models, following the same practices as for the aperiodic EEG data. The model was run separately from aperiodic data, to ensure that aperiodic data was not excluded for participants that did not present a peak in the alpha band.

## 3 Results

### 3.1 Blinding efficacy and outliers

The effectiveness of the single-blind procedure was evaluated based on participants' responses to the post-tES questionnaire (Fertonani et al., 2015). The total amount of self-reported discomfort/sensations associated with stimulation was significantly different between the Stimulation Groups, *U* = 4,895, *p* = 0.014, with higher discomfort reported in the sham (*M* = 2.34, *SD* = 2.76) than in the anodal group (*M* = 2.15, *SD* = 2.00). This difference seems to be mainly driven by the significantly higher intensity reported for *pinching* in the sham group (*M* = 0.73, *SD* = 0.32) than in the anodal group (*M* = 0.28, *SD* = 0.06), *U* = 4688.50, *p* = 0.001, and a marginally higher intensity reported for *burning* in the sham (*M* = 0.82, *SD* = 0.56) as compared to the anodal group (*M* = 0.72, *SD* = 0.36), *U* = 4,619, *p* = 0.045, without any differences for the remaining sensations (all *p*'s > 0.122, see Supplementary Table 1 for further statistical details). The higher discomfort reported in the sham group likely led to a higher estimation of belonging to the active stimulation group in the sham (46%) than in the anodal group (36%). However, and importantly, the guessed active group allocation was not statistically different between Stimulation Groups, χ^2^(2, *N* = 180) = 4.49, *p* = 0.106. The fact that the *active stimulation guess rate* (which has been proposed as more accurate indicator of blinding efficacy that guessing the actual allocated group; Fassi et al., 2023; Fassi and Cohen Kadosh, 2021) was not different across groups, taken together with the evidence for group differences in total discomfort (BF_10_ = 2.03) and pinching (BF_10_ = 0.65) being anecdotal (Lee and Wagenmakers, 2014) at most, led us to conclude that the single-blind procedure was still effective in the present study.

Regarding data filtering and outlier removal, from the EEG data, three participants were excluded during pre-processing, for which less than 80% of the original dataset length remained after visual inspection and artifact removal. From the remaining 177 participants, following the above-described criteria for outlier removal (based on GoF measures), 13 datasets were flagged and discarded [0.61% of total data, i.e., 2124 EEG datasets (participants × pre/post recording × electrodes)] when the FOOOF algorithm was run over the 1–35 Hz range. No datasets were discarded from the data extracted from the 30 to 45 Hz range. From behavioral data, two participants were flagged and excluded from further analyses as their mean accuracy in the EV and/or AV trials was ≤ 50%. Thus, after filtering, 58 participants remained in the single and triple task condition, while 59 remained in the dual task condition.

### 3.2 Neurophysiological effects of HD-tDCS measured through aperiodic parameters and their relationship with task performance

#### 3.2.1 Pre-registered analyses: electrode-specific effects of HD-tDCS on the aperiodic exponent

Against what was hypothesized, no electrode-specific effects of HD-tDCS (i.e., difference between anodes and cathodes, and electrodes part of the stimulation ring vs. only EEG electrodes) were observed on the Δ aperiodic exponent, as evidenced by a non-significant Electrode × Stimulation Condition interaction, for the 1–35 Hz range, *F* < 1, as well as the 30–45 Hz range, *F*_(5, 855)_ = 1.29, *p* = 0.287, η^2^_*p*_ = 0.01. While not pre-registered, performing the same analyses on the Δ aperiodic offset, also revealed no electrode-specific effects of tDCS, given the non-significant Electrode × Stimulation Condition in both frequency ranges, *F*'s < 1. Given the lack of electrode-specific effects across aperiodic parameters, the average across electrodes was used for each measure for further analyses.

**Figure 4:**
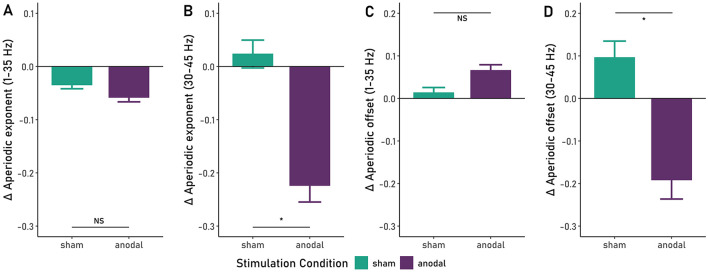
Aperiodic parameters pre-post change (Δ) as a function of Stimulation Condition. **(A)** Δ Aperiodic exponent from the 1 to 35 Hz range, reflecting a slight decrement of the exponent in both conditions; and **(B)** the 30–45 Hz range, reflecting a decrement in the active Stimulation Condition (indicative of a flattened spectral slope); **(C)** Δ Aperiodic offset from the 1 to 35 Hz range, reflecting increments of the offset in both conditions; and **(D)** from the 30 to 45 Hz range, reflecting an increment of broadband power from pre to post in the sham condition, in contrast to a decrement in the active Stimulation Condition. The asterisk (*) denotes significant differences (i.e., *p* < 0.05).

Additionally, from these ANOVAs it was observed that, especially for the 30–45 Hz range aperiodic parameters, the aperiodic exponent decreased from pre- to post-stimulation (i.e., flattening of the spectral slope) with anodal HD-tDCS, evidenced by a significant main effect of Stimulation Condition, *F*_(1, 171)_ = 7.64, *p* = 0.006, η^2^_*p*_ = 0.04 (see Figure 4B). For this frequency range, the same pattern was observed for the aperiodic offset, *F*_(1, 171)_ = 4.99, *p* = 0.027, η^2^_*p*_ = 0.03 (see Figure 4D). For the 1–35 Hz range, these differences were not significant (see Figures 4A, C).

#### 3.2.2 Exploratory analyses: do the aperiodic exponent and offset mediate the effect of HD-tDCS on the executive vigilance decrement?

##### 3.2.2.1 Parallel mediation by Δ aperiodic exponent and offset

To better explore whether the electrode-wide effect of HD-tDCS on aperiodic parameters could provide insight into the behavioral cognitive-load dependent effects reported for this dataset (Hemmerich et al., 2023, 2024), a parallel mediation model was used to pit the aperiodic parameters against each other as mediators of the effect of HD-tDCS on the EV slope in the different task conditions. Given the different effects based on cognitive load (i.e., Task Type), models were run separately for the single, dual, and triple task.

**Figure 5:**
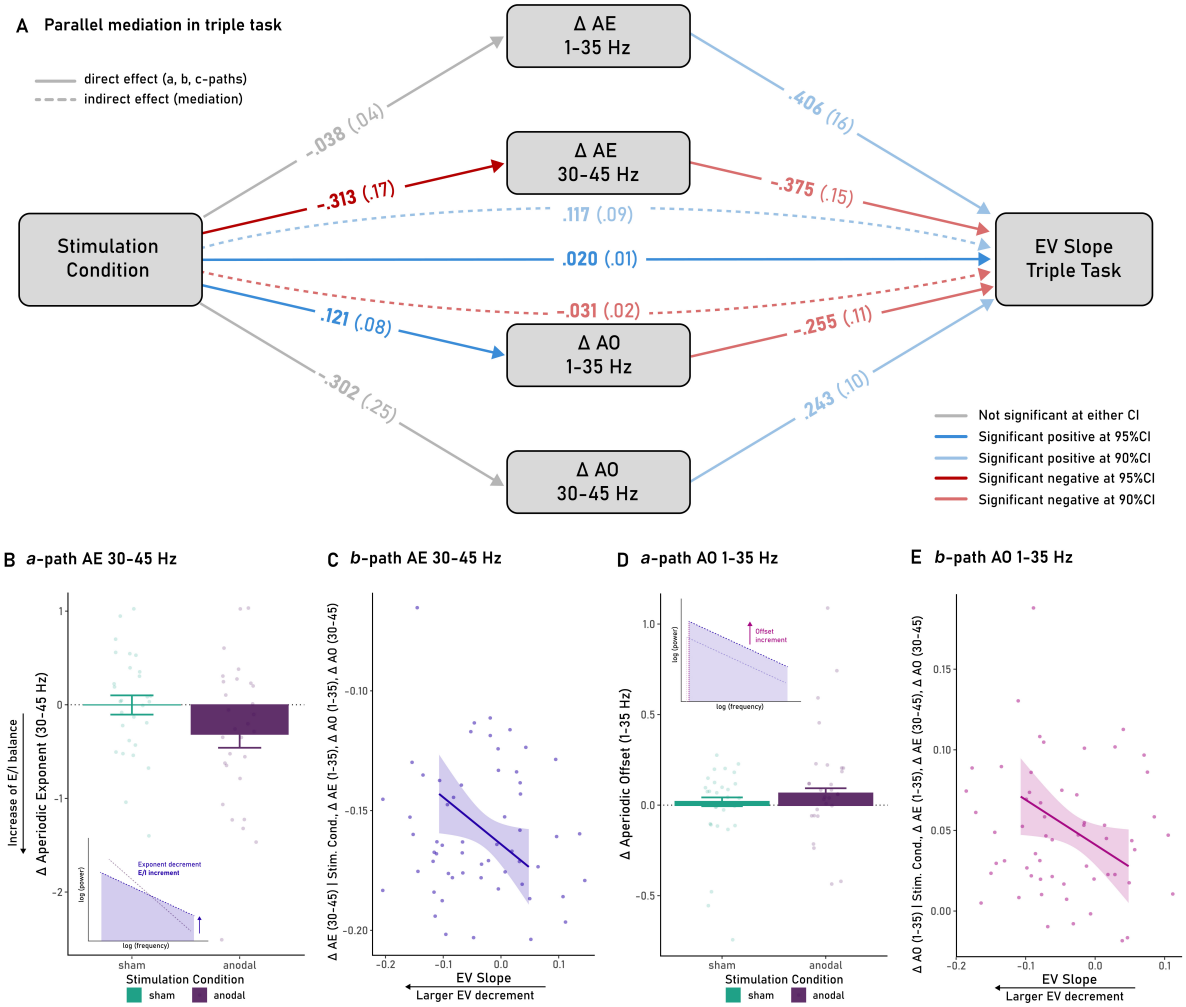
**(A)** Mediation results for aperiodic EEG data in the **triple task**, where two significant indirect effects (represented with dotted curved lines) were observed. **(B)** A greater negative change in the aperiodic exponent (30–45 Hz), indicative of a flattening of the spectral slope, was observed in the group receiving active HD-tDCS; **(C)** which in turn was associated with a mitigated EV decrement. **(D)** Additionally, a significant increment of the aperiodic offset (1–35 Hz), indicative of increased broadband power, was observed in the group receiving active HD-tDCS compared to the sham group; **(E)** which, in turn, was associated with a more pronounced EV decrement. The *b*-paths represented in panels C and E contain the aperiodic variable of interest, whilst controlling for all remaining variables in the model (Stim. Cond., Stimulation Condition; AE, aperiodic exponent; AO, aperiodic offset), as calculated in the model.

**Figure 6:**
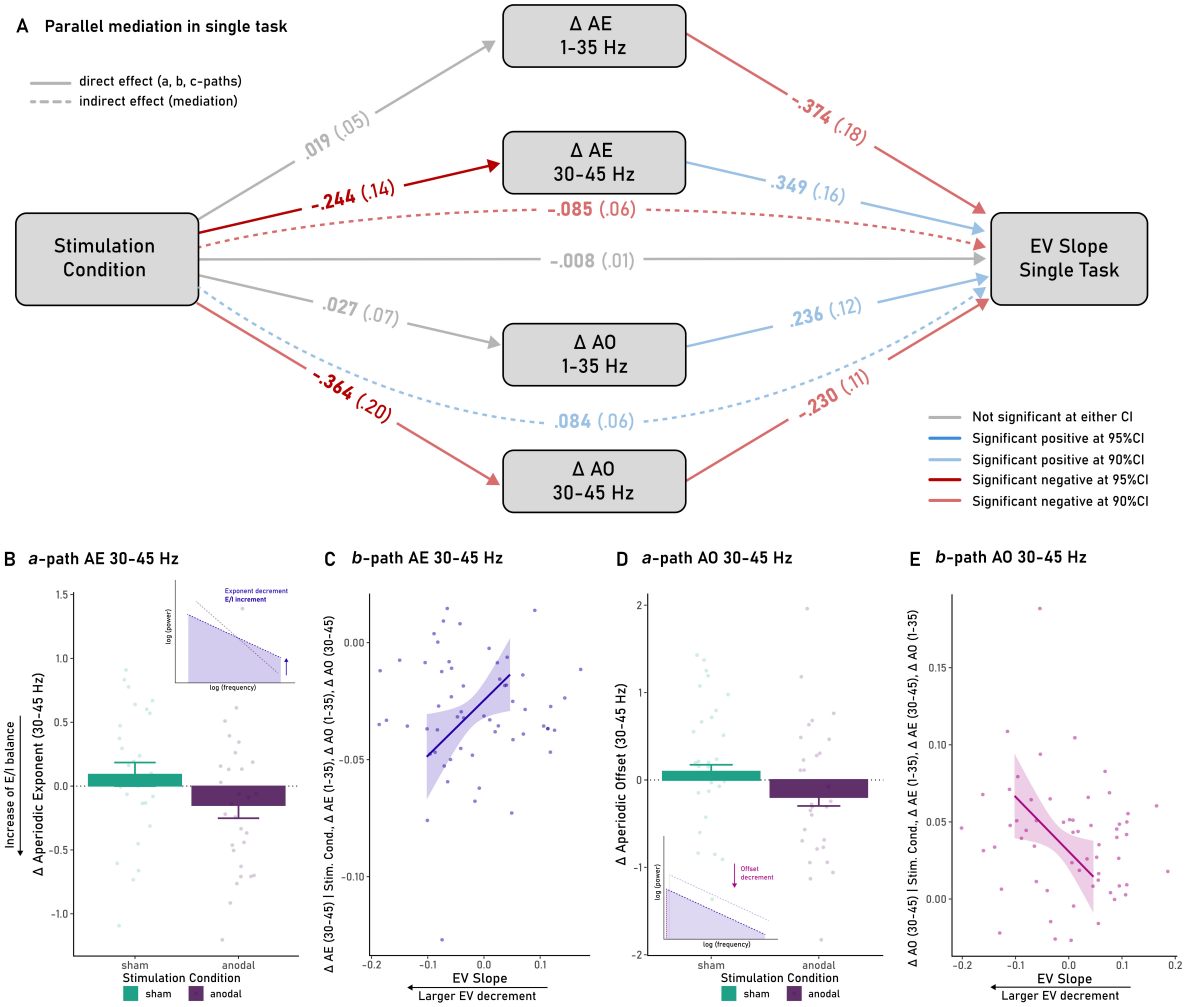
**(A)** Mediation results for aperiodic EEG data in the **single task**, where two significant indirect effects (represented with dotted curved lines) were observed. **(B)** A greater negative change in the aperiodic exponent (30–45 Hz) was observed in the group receiving active HD-tDCS, indicative of a flattening of the spectral slope; **(C)** which in turn was associated with a more pronounced EV decrement. **(D)** Additionally, a significant decrement of the aperiodic offset (30–45 Hz), indicative of decreased broadband power, was observed in the group receiving active HD-tDCS compared to the sham group; **(E)** which, in turn, was associated with a less pronounced or a mitigated EV decrement. The *b*-paths represented in panels C and E contain the aperiodic variable of interest, whilst controlling for all remaining variables in the model (Stim. Cond., Stimulation Condition; AE, aperiodic exponent; AO, aperiodic offset), as calculated in the model.

Results from the parallel mediation models, including aperiodic EEG indices extracted from both frequency ranges (1–35 and 30–45 Hz), revealed a different pattern for the triple and single task (depicted schematically in Figures 5A, 6A). In the *triple task*, participants in the anodal HD-tDCS group showed a significant decrement of the aperiodic exponent (i.e., a flattening of the spectral slope or increased E/I) extracted across the 30–45 Hz frequency range, from pre- to post-stimulation (see Figure 5B and Table 1). This decrement of the aperiodic exponent was, in turn, negatively associated with EV Slope, reflecting that an increased E/I balance was associated with a mitigated EV decrement (see Figure 5C). This result reflected a significant positive indirect effect of HD-tDCS on EV Slope in the triple task, mediated through Δ Aperiodic Exponent (extracted from 30 to 45 Hz) when holding all other mediators constant. Note, however, that while the first effect (*a*-path) was significant at a more stringent CI (95%), the other two effects (*b-*path and indirect effect) were only significant when using a more lenient CI (90%) in a planned manner and thus should be interpret cautiously. Anodal HD-tDCS also significantly increased the offset (extracted from the 1 to 35 Hz range) from pre- to post-stimulation (Figure 5D), which in turn was associated with a more pronounced EV decrement, as reflected by the significant negative indirect effect (see Figure 5E and Table 1). Again, however, the *a*-path was significant at a 95% CI, whereas the *b-*path and indirect effect were only significant under the more lenient CI of 90% (used in an exploratory manner). Furthermore, the significant positive *b*-paths (at CI 90%) for the Δ Aperiodic Exponent (1–35 Hz) and Δ Aperiodic Offset (30–45 Hz) showed that a pre- to post-stimulation increment of these parameters was associated with a mitigated EV decrement. However, these effects occurred in the absence of an effect of HD-tDCS on these variables (see Table 1). Lastly, the application of anodal HD-tDCS was associated with a mitigated EV decrement even when taking into account the indirect effect through the four aperiodic mediators (at CI 90%, planned).

**Table 1:** Mediation model for aperiodic data in triple task.

**Path (effect)**	**Dir. testing**	***b***	***SE***	**95% CI**		**90% CI**	
				**LLCI**	**ULCI**	**LLCI**	**ULCI**
***a-*** **paths: effect of Stimulation Condition on each mediator**							
a_1:_ Δ Exponent (1–35 Hz)	*pl*. (–)	−0.038	0.037	−0.110	0.034	−0.097	0.022
**a**_**2**_**:** **Δ** **Exponent (30–45 Hz)**	*pl*. (–)	**−0.313**	**0.171**	−0.639	0.015	**−0.597**	**−0.033**
**a**_**3**_**:** **Δ** **Offset (1–35 Hz)**	*expl*.	**0.121**	**0.075**	**−0.014**	**0.267**	**0.001**	**0.248**
a_4_: Δ Offset (30–45 Hz)	*expl*.	−0.302	0.250	−0.783	0.195	−0.729	0.091
***b-*** **paths: effect of each mediator on EV slope**							
**b**_**1**_**:** **Δ** **Exponent (1–35 Hz)**	*pl*. (–)	**0.406**	**0.161**	**0.082**	**0.720**	0.135	0.654
**b**_**2**_**:** **Δ** **Exponent (30–45 Hz)**	*pl*. (–)	**−0.375**	**0.151**	**−0.672**	**−0.079**	**−0.619**	**−0.124**
**b**_**3**_**:** **Δ** **Offset (1–35 Hz)**	*expl*.	**−0.255**	**0.105**	**−0.459**	**−0.048**	**−0.423**	**−0.081**
**b**_**4**_**:** **Δ** **Offset (30–45 Hz)**	*expl*.	**0.243**	**0.099**	**0.049**	**0.440**	**0.077**	**0.403**
**Total and indirect effects (Stimulation Condition** > **EV slope)**							
**c (Total effect)**	*pl*. (+)	**0.018**	**0.010**	−0.002	0.038	**0.002**	**0.035**
**c′(Direct effect)**	*pl*. (+)	**0.020**	**0.011**	−0.001	0.042	**0.003**	**0.038**
**Indirect effects (Stimulation Condition** > **mediator** > **EV slope)**							
a_1_b_1_: tDCS 🠞 Δ Exponent (1–35 Hz)	*pl*. (+)	−0.015	0.017	−0.056	0.013	−0.046	0.008
**a**_**2**_**b**_**2**_**: tDCS** 🠞 **Δ** **Exponent (30–45 Hz)**	*pl*. (+)	**0.117**	**0.086**	−0.011	0.312	**0.006**	**0.276**
**a**_**3**_**b**_**3**_**: tDCS** 🠞 **Δ** **Offset (1–35 Hz)**	*expl*.	**−0.031**	**0.021**	−0.076	0.003	**−0.070**	**−0.001**
a_4_b_4_: tDCS 🠞 Δ Offset (30–45 Hz)	*expl*.	−0.074	0.075	−0.254	0.046	−0.216	0.024

N = 58. Dir. testing = directional testing [where: pl. = planned (+ = positive expected effect, – = expected negative effect), expl. = exploratory]. LLCI, lower limit of the CI; ULCI, upper limit of the CI. Significant effects appear in bold.

In the parallel mediation model for the *single task* (shown schematically in Figure 6A and reported in detail in Table 2), a similar excitatory effect of anodal HD-tDCS was observed, as the Δ Aperiodic Exponent (30–45 Hz) significantly decreased from pre- to post-stimulation (see Figure 6B). However, contrary to what was observed for the triple task, in the single task, this decrement of the aperiodic exponent was further associated with a steeper EV decrement (see Figure 6C). This effect was reflected in a significant negative indirect effect of HD-tDCS on the EV Slope, mediated through the Δ Aperiodic Exponent (extracted from 30 to 45 Hz) when holding all other mediators constant. The *a*-path for this effect was significant using a 95% CI, whereas the *b-*path and indirect effect reached significance only under a 90% CI in a planned manner; these latter results should therefore be interpreted cautiously. Notably, in the single task a positive indirect effect of HD-tDCS on the EV Slope was observed through the Δ Aperiodic Offset (extracted from 30 to 45 Hz), as here, anodal HD-tDCS induced a decrement of the offset, which in turn was associated with a less pronounced or a mitigated EV decrement (Figures 6D, E). However, while the *a*-path was significant at a 95% CI, whereas the *b-*path and indirect effect were only significant under the more lenient CI of 90% (exploratory). Additionally, a significant negative *b*-path (only at a 90% CI, planned) for Δ Aperiodic Exponent (1–35 Hz) and a significant positive *b*-path (only at a 90% CI, exploratory) for Δ Aperiodic Offset (1–35 Hz) were observed, however, not attributed to any effect of HD-tDCS on these indices.

**Table 2:** Mediation model for aperiodic data in single task.

**Path (effect)**	**Dir. testing**	***b***	***SE***	**95% CI**		**90% CI**	
				**LLCI**	**ULCI**	**LLCI**	**ULCI**
***a*** **-paths: effect of Stimulation Condition on each mediator**							
a_1_: Δ Exponent (1–35 Hz)	*pl*. (–)	0.019	0.049	−0.080	0.117	−0.063	0.096
**a**_**2**_**:** **Δ** **Exponent (30–45 Hz)**	*pl*. (–)	**−0.244**	**0.135**	−0.508	0.010	**−0.454**	**−0.014**
a_3_: Δ Offset (1–35 Hz)	*expl*.	0.027	0.073	−0.113	0.165	−0.094	0.150
**a**_**4**_**:** **Δ** **Offset (30–45 Hz)**	*expl*	**−0.364**	**0.196**	−0.742	0.006	**−0.676**	**−0.041**
**b-paths: effect of each mediator on EV slope**							
**b**_**1**_**:** **Δ** **Exponent (1–35 Hz)**	*pl*. (+)	**−0.374**	**0.179**	**−0.750**	**−0.043**	−0.690	−0.096
**b**_**2**_**:** **Δ** **Exponent (30–45 Hz)**	*pl*. (+)	**0.349**	**0.161**	**0.061**	**0.713**	**0.107**	**0.645**
**b**_**3**_**:** **Δ** **Offset (1–35 Hz)**	*expl*.	**0.236**	**0.118**	**0.017**	**0.493**	**0.055**	**0.444**
**b**_**4**_**:** **Δ** **Offset (30–45 Hz)**	*expl*.	**−0.230**	**0.107**	**−0.473**	**−0.044**	**−0.429**	**−0.074**
**Total and indirect effects (Stimulation Condition** > **EV slope)**							
c (Total effect)	*pl*. (–)	−0.010	0.008	−0.025	0.006	−0.023	0.003
c′ (Direct effect)	*pl*. (–)	−0.008	0.008	−0.024	0.008	−0.021	0.006
**Indirect effects (Stimulation Condition** > **Mediator** > **EV slope)**							
a_1_b_1_: tDCS 🠞 Δ Exponent (1–35 Hz)	*pl*. (–)	−0.007	0.021	−0.055	0.030	−0.045	0.021
**a**_**2**_**b**_**2**_**: tDCS** 🠞 **Δ** **Exponent (30–45 Hz)**	*pl*. (–)	**−0.085**	**0.064**	−0.242	0.004	**−0.210**	**−0.003**
a_3_b_3_: tDCS 🠞 Δ Offset (1–35 Hz)	*expl*.	0.007	0.020	−0.031	0.048	−0.022	0.040
**a**_**4**_**b**_**4**_**: tDCS** 🠞 **Δ** **Offset (30–45 Hz)**	*expl*.	**0.084**	**0.062**	−0.002	0.236	**0.005**	**0.208**

N = 58. Dir. testing = directional testing [where: pl. = planned (+ = positive expected effect, – = expected negative effect), expl. = exploratory]. LLCI, lower limit of the CI; ULCI, upper limit of the CI. Significant effects appear in bold.

In contrast to these results, in the dual task, no paths were significant for the parallel mediation model, including all aperiodic variables (see Supplementary Table 2 for full results).

##### 3.2.2.2 Single mediation by Δ aperiodic exponent and offset

**Figure 7:**
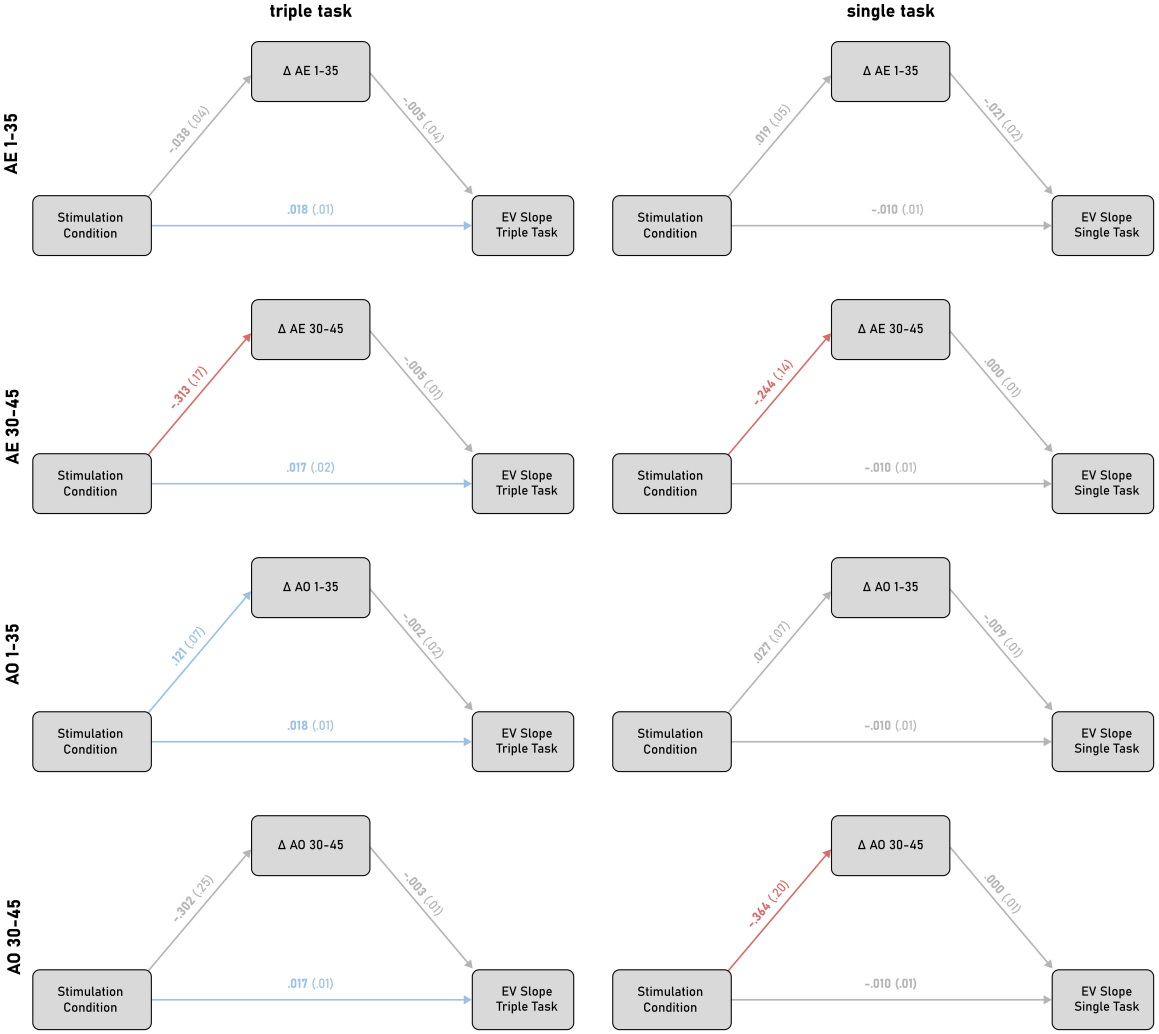
Single mediation models for the triple (left column) and single (right column), for each aperiodic index introduced as a mediator separately. AE, aperiodic exponent; AO, aperiodic offset.

Given that some of the mediators included in the parallel mediation model were correlated or highly correlated (see Supplementary Table 3), single mediation models, including only one of the aperiodic parameters at a time were also performed. In contrast to what was observed in the parallel mediation models, no significant mediation (indirect effects) was observed when each aperiodic variable is introduced separately into single mediation models testing the direct and indirect effects of Stimulation Condition on EV Slope, as summarized in Figure 7 (see Supplementary Tables 4–11 for the full result tables for each model). While the *a*-paths that were significant in the parallel models remain significant in these individual models, the effects observed in the *b*-paths, and, as a consequence, the indirect effects, were not observed.

### 3.3 Can the pre-stimulation (on-task) aperiodic exponent predict HD-tDCS outcomes?

#### 3.3.1 Pre-registered analysis: binomial generalized mixed effects model on baseline data and accuracy in EV trials

**Figure 8:**
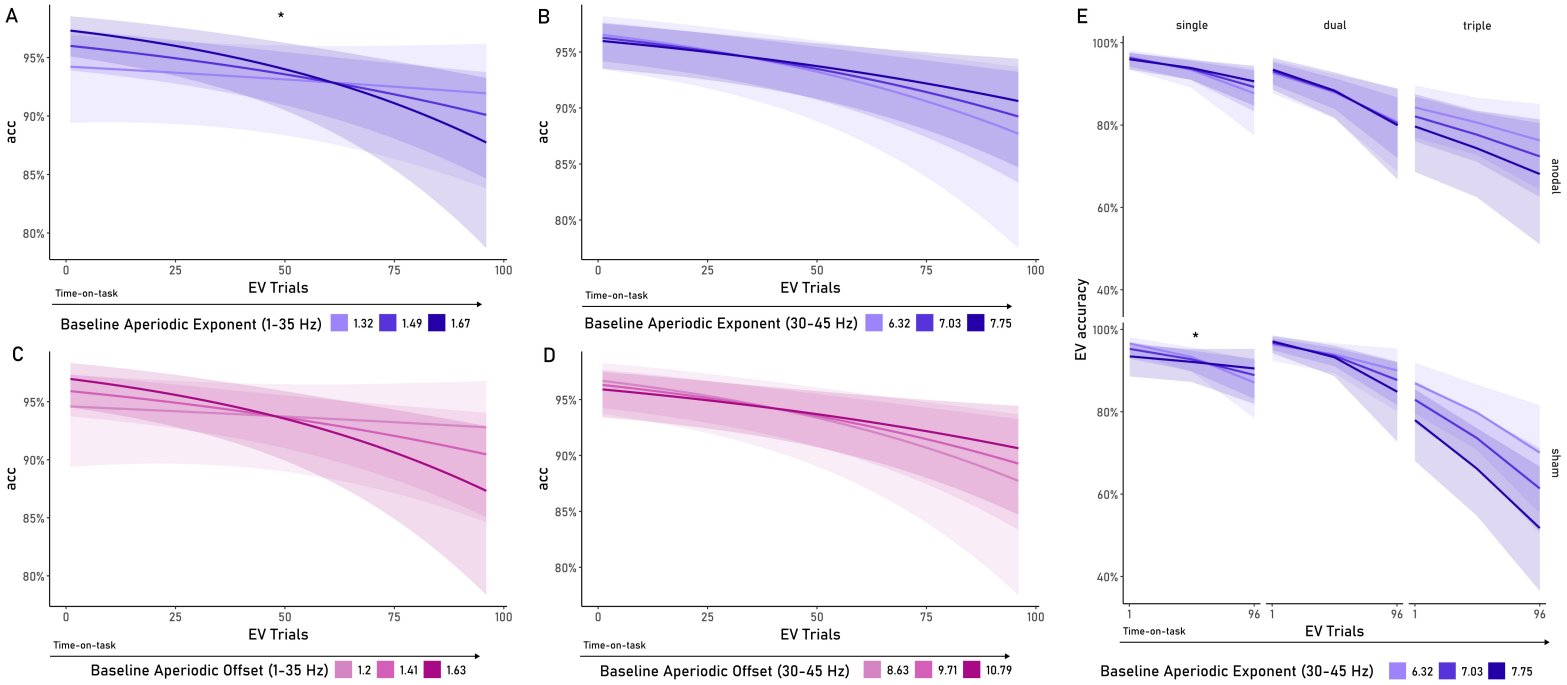
**(A)** Predicted accuracy for EV trials (across all three Task Types) as a function of time-on-task (EV Trials) and Baseline Aperiodic Exponent and Offset (splitting the continuous variables into representative steps, from lower to higher values), showing that: Higher values of the Baseline Aperiodic Exponent extracted from the 1 to 35 Hz range, predicted a steeper decrement of accuracy in EV trials with time-on-task; **(B)** whereas, this relationship was not observed with the Baseline Aperiodic Exponent extracted from the 30 to 45 Hz range. **(C)** Higher values of the Baseline Aperiodic Offset extracted from the 1 to 35 Hz range, predicted a steeper decrement of accuracy in EV trials with time-on-task; **(D)** whereas this relationship was not observed with the Baseline Aperiodic Offset extracted from the 30 to 45 Hz range. **(E)** Predicted accuracy for EV trials as a function of Task Type, Stimulation Condition, time-on-task (EV Trials), and Baseline Aperiodic Exponent (30–45 Hz), showing the significant interaction in the single task, sham condition, where a flatter spectral slope predicted more stable EV performance. The asterisk (*) denotes significant differences (i.e., *p* < 0.05).

A further pre-registered question was whether baseline aperiodic EEG parameters (in this case, from on-task, pre-stimulation EEG data) could predict or determine the effects of HD-tDCS on the EV decrement across different Task Types. To answer this question, the pre-registered binomial GLM on accuracy data from EV trials (96 trials), showed a significant EV Trials × Baseline Aperiodic Exponent (1–35 Hz) interaction, χ^2^_(1)_ = 4.15, *p* = 0.042, reflecting that steeper EV decrements (lower accuracy in EV trials with time-on-task) were associated with higher values of the aperiodic exponent (1–35 Hz) at baseline (i.e., a steeper spectral slope), whereas participants with lower Baseline Aperiodic Exponent values (i.e., a flatter spectral slope) showed more stable performance across time-on-task, as shown in Figure 8A. Notably, this effect was broadly observed across Task Type and Stimulation Condition. On the other hand, this effect was not observed for the 30–45 Hz range, as the EV Trials × Baseline Aperiodic Exponent (30–45 Hz) interaction was not significant, χ^2^_(2)_ = 0.75, *p* = 0.686, showing no association with baseline values (Figure 8B).

For the Baseline Offset, the EV Trials × Baseline Aperiodic Offset (1–35 Hz) interaction showed a trend toward the same effect observed for the exponent, χ^2^_(1)_ = 3.13, *p* = 0.077, suggesting that larger values of the offset at baseline in the 1–35 Hz range could be associated with a steeper EV decrement (i.e., a decrement in accuracy in EV trials) with time-on-task, as shown in Figure 8C. The same analysis on the Baseline Aperiodic Offset from the 30 to 45 Hz range was not significant, χ^2^_(1)_ = 1.16, *p* = 0.281 (Figure 8D).

However, follow-up inspection of specific model coefficients revealed that for participants receiving sham tDCS and performing the single task, baseline aperiodic activity in the 30–45 Hz range interacted with EV accuracy across EV Trials. In this condition, participants with higher Baseline Aperiodic Exponent in the 30–45 Hz range (i.e., a steeper pre-stimulation spectral slope, indicative of higher inhibition) showed a significantly more stable EV performance across time-on-task (*b* = 0.013, *SE* = 0.01, *z* = 2.01, *p* = 0.045), as shown in Figure 8E. A similar trend was observed for the Baseline Aperiodic Offset in the 30–45 Hz range, which showed a marginal effect in the same direction (*b* = 0.013, *SE* = 0.01, *z* = 1.97, *p* = 0.050). While these effects did not emerge at the omnibus level, their presence in this specific subgroup suggests possible task- and stimulation-dependent modulations of the EV decrement determined by baseline high-frequency aperiodic features.

#### 3.3.2 Exploratory analysis: moderation by baseline aperiodic data

Given the slightly contradictory results obtained from the pre-registered approach, the same question regarding baseline aperiodic parameters was re-explored through moderation analyses. Models including the direct effect of Stimulation Condition on EV Slope, and both Task Type and Baseline values of either aperiodic index (exponent and offset from the 1 to 35 and 30 to 45 Hz ranges), revealed no significant moderated moderation (i.e., triple interaction of Stimulation Condition × Task Type × Baseline Aperiodic Exponent/Offset) on the EV Slope as an outcome. For statistical details, see Supplementary Tables 15–18.

Notably, although the hypothesized moderated moderation (i.e., a three-way interaction between Stimulation Condition, Baseline Exponent, and Task Type) was not observed, a significant interaction emerged between Baseline Aperiodic Exponent (30–45 Hz) and Task Type on EV Slope (Supplementary Table 15). Specifically, higher values of Baseline Aperiodic Exponent (in the 30–45 Hz range) were associated with a less negative EV Slope (i.e., a smaller EV decrement) in the single-task condition (used as the reference in the model, Baseline Aperiodic Exponent, *b* = 0.011, *SE* = 0.005, 90% CI [0.000, 0.017], 95% CI [−0.003, 0.020]), but this relationship was significantly reduced when compared to the dual-task (*b* = −0.023, *SE* = 0.011, 95% CI [−0.046, −0.002]) and triple-task (*b* = −0.023, *SE* = 0.011, 95% CI [−0.046, −0.001]) conditions. These results suggest that baseline neural excitability (as indexed by the aperiodic exponent) moderates the effect of cognitive load on EV performance (independent of stimulation), with stronger predictive value under lower cognitive load conditions.

## 4 Discussion

The present study aimed to explore the potential role of aperiodic (and periodic) EEG markers that explain the efficacy of HD-tDCS in mitigating the decrement of EV. A recent spike in the interest toward aperiodic EEG activity has shown its potential to index task-induced changes in the neural balance between excitation and inhibition (E/I balance, Gao et al., 2017; Lendner et al., 2020), serving as a putative marker to better understand the effects of interventions with tES (Krause et al., 2013; Sheffield et al., 2020; van Bueren et al., 2023). This approach has not yet been explored in relation to the effects of tDCS on the vigilance decrement. In the present study we observed an overall reduction in the aperiodic exponent (indicating an increased E/I balance) in the 30–45 Hz range in response to HD-tDCS over the rPPC in the triple and single tasks. This decrement of the aperiodic exponent in the 30–45 Hz range mediated the effect of HD-tDCS on the EV decrement, with opposite effects in the triple and single task conditions: explaining the beneficial effects of HD-tDCS in the triple task, and the detrimental effects in the single task. However, in each task condition, an opposite mediation effect was observed with the aperiodic offset (from different frequency ranges for each task), which potentially obscured or suppressed the effects observed for the exponent. Lastly, contrary to what was hypothesized, baseline values in either aperiodic or periodic data did not moderate the effect of the HD-tDCS application over the rPPC on the EV decrement. However, broader associations between baseline aperiodic values and performance were observed, independent of stimulation. The implications as well as limitations of these results will be discussed in more detail in the following section.

Regarding the direct effect of the application of the HD-tDCS protocol on aperiodic EEG measures, we had established electrode-specific hypotheses (based on the polarity of the electrodes conforming the HD-tDCS ring), expecting a decrement of the aperiodic exponent to reflect an increase of E/I (flattening of the spectral slope) under the anode and the opposite effect under the cathodes. However, no such specificity was observed in pre-post stimulation changes. Instead, we observed that the overall aperiodic exponent extracted from a higher frequency range (30–45 Hz) more accurately reflected the increment of E/I (flattening of the spectral slope) with active stimulation compared to the exponent extracted from a lower range (1–35 Hz). The non-specific effect as a function of electrode might be explained by the use of an HD-tDCS protocol in the current study. The use of the concentric 4 × 1 stimulation ring has actually shown to produce a skewed e-field in favor of the polarity of the central electrode (Alam et al., 2016), peaking under the central electrode and dissipating toward the surrounding return electrodes (Edwards et al., 2013). While polarity-dependent effects on the aperiodic exponent have been reported with traditional tDCS montages (Kang et al., 2025), the almost “unipolar” electric field generated from the montage used in this study might have been more likely to produce wide-spread effects on cortical excitability in the same direction. The fact that the aperiodic exponent extracted from the higher frequency range (30–45 Hz) better captured the expected effect of HD-tDCS (flattening of spectral slope, indicative of increased E/I balance) may be due to broadband power measures from higher frequency ranges being less obscured by oscillatory activity in lower frequencies (He, 2014). Notably, a study that served as a relevant foundation for interpreting the aperiodic exponent as an indirect measure of the E/I balance used a relatively similar frequency range (30–70 Hz) to the higher range in the present study (Gao et al., 2017). Furthermore, Lendner et al. (2020) explored different frequency ranges (ranging across 1–20, 1–40, and 30–45 Hz fits), and concluded that the aperiodic exponent extracted from the 30 to 45 Hz range better predicted different states of arousal across wakefulness, anesthesia, and different sleep stages. Höhn et al. (2024) showed that the aperiodic exponent from a narrowband range (30–45 Hz) better predicted performance in a Go-NoGo task than the exponent from a broader range (1–45 Hz). Similarly, Pei et al. (2023), compared different aperiodic fits (1–25 and 26–90 Hz) and power measures from canonical frequency bands, and observed that the aperiodic exponent and offset extracted from the higher frequency range showed the greatest association with cognitive load-dependent variations in performance.

It must be noted that, given that EEG power spectra only follow a 1/*f*-like structure, and not a true 1/*f* structure (Donoghue, 2025), due to the existence of spectral plateaus at both low and high frequencies (Gerster et al., 2022), the frequency range from which aperiodic parameters are extracted will influence the parameters' values, and potentially, their functional interpretation. Furthermore, aperiodic components extracted from higher frequency ranges potentially scale more directly with asynchronous firing rates (Gao et al., 2017), providing a potential mechanistic explanation for the heightened sensitivity to the E/I balance changes. In contrast, lower frequencies may be more strongly shaped by prominent peaks of periodic (oscillatory) peaks that could non-linearly distort the spectral slope, reducing the sensitivity of aperiodic parameters in capturing changes in broadband neural activity (Brake et al., 2024). Although frequencies above ~30 Hz are more susceptible to being conflated with myogenic activity (Muthukumaraswamy, 2013), the above-mentioned emerging evidence of a link between high-frequency aperiodic parameters and different cognitive processes, supports the idea that these indices may convey meaningful information on neural excitability. Taken together, these considerations highlight that (i) the choice of frequency range critically shapes both the numerical values and functional interpretation of aperiodic parameters, and (ii) future research should more systematically investigate whether aperiodic measures derived from distinct spectral ranges reflect partly different physiological processes or exhibit differential sensitivity to neuromodulatory interventions such as HD-tDCS.

**Figure 9:**
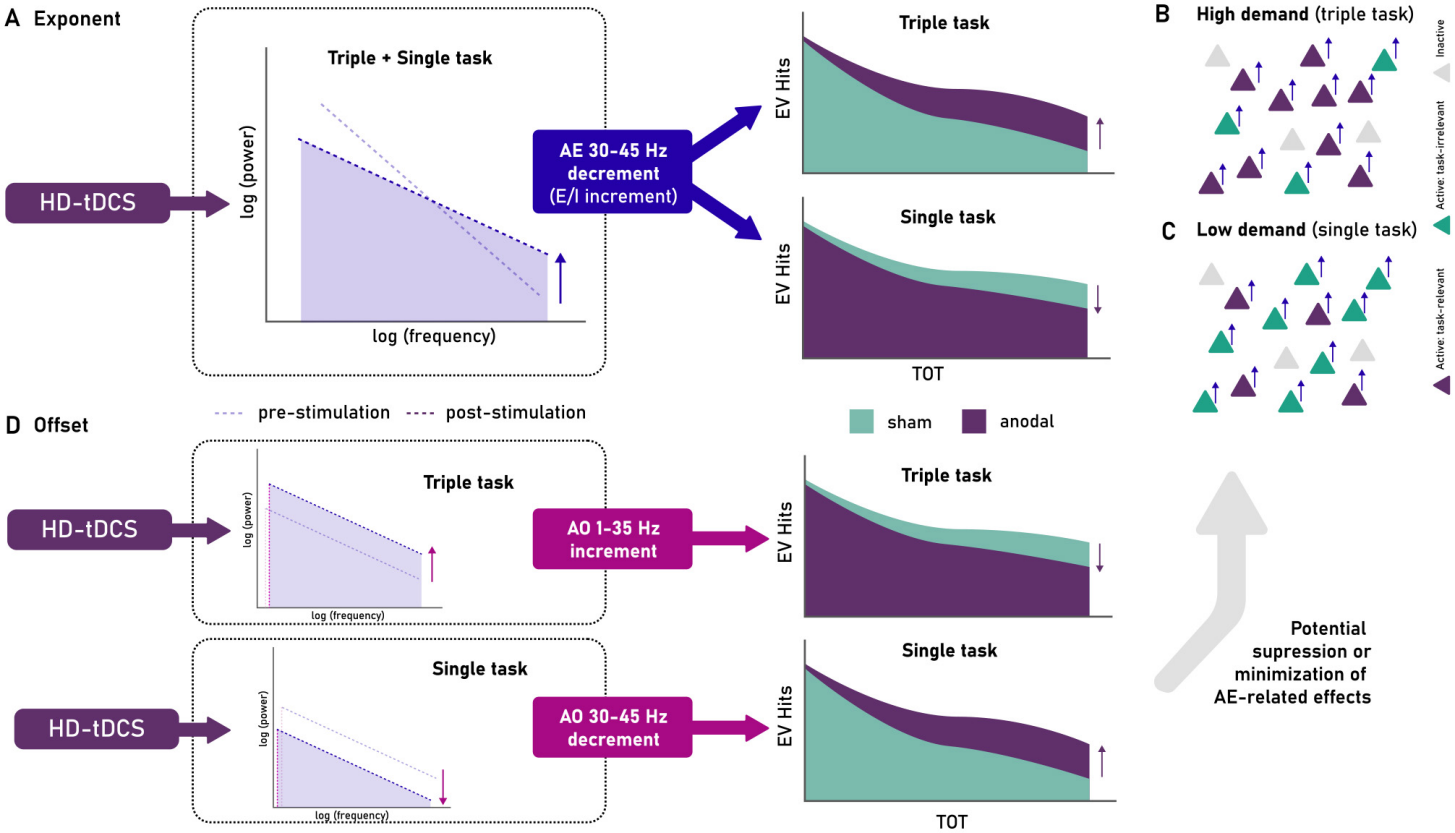
Summary of results and a suggested explanatory model. **(A)** Summary of results of the effect of HD-tDCS on the EV decrement in triple and single task conditions mediated through the decrement of the aperiodic exponent, which reflects an increase of E/I. **(B)** Putative task-dependent effect of tDCS on EV performance mediated by the aperiodic exponent: near-threshold neurons/connections are those that are activated by task-relevant (purple) or task-irrelevant (green) processes, which are more likely to be further excited by the tDCS-induced increment of excitability, compared to inactive neurons (gray). In the triple task, a higher cognitive demand engages a larger number of task-relevant neurons/connections, which when further excited by tDCS leads to improved performance. **(C)** The single task can be achieved with a more efficient processing: potentially requiring less active task-relevant neurons/connections for producing adequate performance. Therefore, task-irrelevant areas might be more active, engaging in other non-related activities (such as mind-wandering). If this pattern of activity is further excited by tDCS, the further facilitation of task-irrelevant processes might lead to a detrimental effect on performance. **(D)** Effects of HD-tDCS on the aperiodic offset, which according to mediation models suppresses or minimizes the relationship observed with the aperiodic exponent. AE, aperiodic exponent; AO, aperiodic offset; TOT, time-on-task.

While the exploration of the direct effect of tDCS on the aperiodic parameters is of great interest, as to further understand the intricate neurophysiological effects of tDCS, the design of the present study is not optimal for this purpose. Given that the EEG data analyzed in the present study was collected during the first and last minutes of the behavioral task, the neurophysiological and task-related effects on EEG cannot be measured independently and are likely to show an interaction. This is further supported by the fact that, whilst the same HD-tDCS protocol induced the same effect in the triple and single tasks on the aperiodic exponent extracted from the 30 to 45 Hz range, it did not produce a significant effect in the dual task condition. Therefore, a look into the overall relationship across the HD-tDCS-EEG-behavior axis might be more appropriate. The mediation analyses conducted to explore whether any of the aperiodic indices could provide a potential mechanistic explanation of the effects of HD-tDCS on behavior revealed mixed results. When pitting the different aperiodic indices against each other in a parallel mediation model, to see which one (if any) revealed a potential indirect effect, we observed an opposite pattern between the triple and the single task conditions, as schematically depicted in Figure 9. As discussed in the prior points, for the triple and single task a significant negative *a*-path was observed for the aperiodic exponent extracted from the 30 to 45 Hz range (i.e., a flattening of the power spectra from pre- to post-task with active HD-tDCS, as shown in Figure 9A). However, interestingly, this neurophysiological effect had the opposite effect on behavior in each task condition. In the triple task condition, the decrement of the aperiodic exponent predicted a mitigated EV decrement (i.e., a flattening of the spectra or an increased E/I balance was associated with better performance). On the other hand, in the single task condition, the same decrement of the aperiodic exponent predicted a more pronounced EV decrement (i.e., a flattening of the spectra or an increased E/I balance was associated with worse performance). These results are important as they support a previous theoretical framework that posits that tES needs to drive an optimal change in E/I balance (Krause et al., 2013), but highlight that this needs to be tailored to the task at hand. However, it must be noted that the relationship between the aperiodic exponent and performance, as well as the resulting mediation effects, although consistent with predictions, were only significant under directional hypothesis testing. Thus, while aligning with existing literature, further research and replication is needed to confirm these effects.

These results highlight the sensitivity of the effects of tDCS in interaction with task-evoked patterns of brain activity. The triple task, while demanding, is not considered to be overtaxing, and therefore, increasing excitation in task-relevant areas (Figure 9B), may facilitate performance and mitigate the drops in detection accuracy with time-on-task. The single task condition, on the other hand, whilst presenting overall better and less steep performance decrements with time-on-task, may produce a more mixed pattern of brain activity. If we assume, as proposed by Thomson et al.'s (2015) resource-control account, that available resources are constant, the single task would lead to task-relevant processes to be working in parallel with other non-related operations (i.e., mind-wandering). In that case, further exciting this pattern of activity with tDCS, may further promote task-unrelated thoughts which would lead to the observed detrimental behavioral effects (Figure 9C). Nonetheless, this mechanistic account should be seen as a speculative interpretation rather than a tested causal model, and future work will be needed to empirically validate these assumptions. Such non-linear state-dependent effects of non-invasive brain stimulation (NIBS) have been attributed to stochastic resonance, suggesting that processes operating at threshold levels may benefit from an optimal level of noise, compared to conditions of absent or excessive noise (Abrahamyan et al., 2011; Miniussi et al., 2013). While this conceptualization of certain processes benefiting from the input of a certain level of noise to improve information processing has been more popular in considering the effects of transcranial magnetic stimulation (Miniussi et al., 2013) and tRNS (Fertonani and Miniussi, 2017), recent evidence points toward its potential application to also understand behavioral outcomes of tDCS (Benwell et al., 2015; Bestmann et al., 2015). As the degree of noise introduced into the system by tDCS might be lower than with the aforementioned techniques, the dependence on the endogenous signal-to-noise ratio, which could be induced by different task types, could be all the more relevant. That is, given the relatively lower level of noise introduced by tDCS, an optimal interaction is found with the more demanding (triple) task, as task-induced noise levels are elevated, thus allowing the additional introduction of noise from tDCS to achieve an improved signal-to-noise ratio for adequate information processing. This can be further tied in with the conceptualization of the aperiodic exponent as a measure of noise in underlying neural networks (He et al., 2010; Voytek and Knight, 2015), where a flattening of the spectral slope is an approximation toward the function of white noise. The input of noise via tDCS (i.e., flattening of the spectral slope) observed in the present study, may, in that case, induce an optimal signal-to-noise ratio in the triple task condition based on the underlying activation induced by the task, whereas the addition of noise in the single task interacts in a detrimental manner with ongoing brain activity.

A further interesting—yet preliminary—finding obtained from the parallel mediation model is that the aperiodic offset showed trend-level exploratory indirect effects that are, again, opposite for the triple and single task, and additionally opposite to the indirect effect observed for the aperiodic exponent within each task type (summarized in Figure 9D). In the triple task, a negative indirect effect is observed, as active HD-tDCS was associated with an increment of the pre-post stimulation aperiodic offset (extracted from the 1 to 35 Hz range); which in turn was associated with a steeper EV decrement. This result suggests that in parallel to the beneficial effect of HD-tDCS on performance explained through the aperiodic exponent extracted from the higher frequency range, a detrimental effect is taking place, explained through the increment of the aperiodic offset from the lower frequency range with active HD-tDCS. Given that overall, the active HD-tDCS protocol produces a mitigated EV decrement, the effect on the aperiodic exponent might be stronger, but its efficacy may be reduced due to the additional effects on the aperiodic offset. For the single task, on the contrary, active HD-tDCS was associated with a decrement of the pre-post stimulation aperiodic offset (extracted from the 30 to 45 Hz range); which in turn was associated with a mitigated EV decrement. Thus, here the contrary opposing indirect effects through aperiodic parameters seem to be more balanced, leading to the slightly detrimental direct effect of HD-tDCS on behavior that does not reach significance. However, these effects should be taken with particular caution due to their exploratory nature. Nonetheless, these results underscore the importance of considering the aperiodic offset, which is often not reported or analyzed in studies investigating aperiodic features of EEG data, yet it has shown higher predictive power than the exponent in some cases (Turri et al., 2023). Furthermore, these findings seem to support the separate nature of the aperiodic exponent and offset (Donoghue et al., 2020b). In line with our results, Ouyang et al. (2020) report opposing behavioral correlates (in this case with processing speed) with the exponent as compared to the offset.

The above-outlined interpretation of the parallel mediation results provides a promising explanation for how tDCS effects vary with different tasks and underscores the importance of choosing tasks with the right level of cognitive demand to pair with online tDCS. However, it is important to also consider some limitations to the reliability of these findings, namely, that results did not replicate when introducing each aperiodic variable separately into a single mediation model. Whilst the *a*-paths remained significant as to what was observed in the parallel mediation models, the effects of each aperiodic variable on the EV slope, and with it, also the mediated effects of tDCS via these variables, disappeared. Two potential explanations emerge for this discrepancy between the two analysis approaches. First, the way the relationship between the different aperiodic parameters and the behavioral outcomes, as well as the mediated effects, are explored in the parallel mediation model, controls all other variables in each step. Therefore, the presence of potential conflicting effects (as suggested by the opposite effects in the aperiodic exponent and offset) may obscure the role of each variable when investigating its mediating role individually, leading to a suppression effect (MacKinnon et al., 2000). A second alternative explanation to the opposite findings between the parallel and single mediation models is the potential overlap between the different mediators, as the frequency fits are slightly overlapped (over a 5 Hz range) and the exponent and offset may share a certain dependency that could affect the model output. The parallel mediation model operates under the assumption that the mediators do not causally influence each other, whilst they are allowed to correlate (Hayes, 2022). Taken together, the divergence between the parallel and single mediator models raises the possibility that the observed effects are unstable or highly sensitive to model specification.

Contrary to what was observed for aperiodic data, no significant mediation was observed for the pre-post stimulation change in alpha power (results reported in Supplementary material). Notably, a significantly reduced alpha power increment with time-on-task was observed in the triple task condition, as compared to the single and dual task conditions. This finding in the triple task condition, therefore, replicates prior findings (Hemmerich et al., 2023), observed when analyzing alpha power without parametrizing the power spectra (i.e., measuring alpha power including potential shifts in aperiodic data, as compared to only measuring alpha as the peak above the aperiodic exponent). This result further points toward the fact that alpha power is a useful estimator of time-on-task effects in high-demand tasks, likely reflecting an increased need for the inhibition of task-irrelevant information (Clayton et al., 2015), in order to keep cognitive resources on task-relevant processes. However, the impact of HD-tDCS on alpha power was not related to behavioral outcomes in the present study, thereby offering no mechanistic insight into the effectiveness or ineffectiveness of the HD-tDCS intervention. The role of alpha oscillations in explaining tDCS efficacy may be found when considering it as part of an interactive mechanism with other processes but not on its own; as suggested, for example, by the Alpha_parietal_/Gamma_frontal_ index reported by Hemmerich et al. (2023). Notably, despite an interest in gamma power in relation to these prior findings, the periodic contribution of this frequency range could not be explored further due to a lack of sufficient observations (i.e., many participants did not have any peaks above the aperiodic exponent in the 30–45 Hz frequency range). This finding may point to the fact that, what was observed as changes in gamma power as reported in Hemmerich et al. (2023), may in fact be a shift in broadband power, without any real periodic contribution. This aligns with the potential interpretation of the aperiodic offset as suggested by Manning et al. (2009). On the other hand, the significant flattening of the power spectrum with HD-tDCS in the 30–45 Hz range, and the trend in the same direction across the 1–35 Hz range, albeit not statistically significant, suggests a global flattening of the spectral slope across the full 1–45 Hz range. This pattern suggests that the Alpha_parietal_/Gamma_frontal_ index used by Hemmerich et al. (2023), derived from the non-parametrized spectrum, may have indirectly captured changes in the aperiodic exponent. Specifically, under active HD-tDCS, the Alpha_parietal_/Gamma_frontal_ index decreased, reflecting reduced alpha power and increased gamma power. Such a shift aligns with the characteristics of a flattened aperiodic slope—namely, reduced power at lower frequencies and elevated power at higher frequencies. These findings underscore the importance of using parametrized spectral measures to more precisely isolate and interpret the contributions of aperiodic activity to neurophysiological and behavioral outcomes.

Apart from exploring the pre-post changes in aperiodic and periodic data in search of a mechanistic explanation, we further explored whether the effect of HD-tDCS on the EV decrement was moderated by baseline values of these aperiodic and periodic. Against what was hypothesized, no significant moderation for either aperiodic component was observed. The use of baseline measures as a means of predicting individual responsiveness to tES has shown promising results, both with behavioral (Brosnan et al., 2018; Gan et al., 2022; Looi et al., 2016) and neurophysiological measures (Filmer et al., 2019; Sheffield et al., 2020; van Bueren et al., 2023). The predictions for the present study had been based on promising findings observed with tRNS interventions (Sheffield et al., 2020; van Bueren et al., 2023). The fact that no moderating effects were observed in the present study could, therefore, be explained by the use of a different tES technique, by the outcome being measured on a different cognitive process, the use of on-task baseline data compared to rs-EEG data, or other methodological differences between studies. However, broader associations between baseline values of aperiodic data and EV performance were observed, independent of the application of tDCS. Through a more fine-grained approach (inspecting trial-by-trial data), it was observed that both larger aperiodic exponent and offset values at baseline from the 1 to 35 Hz range predicted steeper accuracy declines in EV trials over time, whereas lower values were associated with more stable performance. These results suggest that a steeper spectral slope (lower E/I balance) and higher broadband power in the initial moments of the task, create a greater vulnerability to time-on-task effects. However, a different inspection of results yielded a condition-specific effect, showing that in low-demand conditions without stimulation (i.e., single task with sham tDCS), larger baseline values of the exponent from the 30 to 45 Hz range predicted a more stable performance in EV trials. Potentially in line with findings from Ouyang et al. (2020), performance in a relatively simple task may benefit from higher levels of inhibition, which might aid in inhibiting task-irrelevant thoughts. In addition, when approaching the same data with a more summarized behavioral outcomes measure (EV Slope across task blocks), while still not observing HD-tDCS-related effects, it was observed that specifically for the single task condition, a higher baseline exponent from the 30 to 45 Hz range was associated with a mitigated EV decrement. The discrepancies between the pre-registered and the exploratory analyses may reflect differences in analytic approach (trial-by-trial vs. summary slope). However, overall, the results still suggest that individual differences in baseline aperiodic EEG dynamics may shape susceptibility to the EV decrement in a broader manner (i.e., potentially independently of tDCS effects).

While some specific limitations pertaining to the findings in the present study have already been discussed above, some more global limitations must be taken into account. First, the type of EEG data used in the present study, whilst providing more direct information on task-related processing, is also more difficult to interpret as it does not allow an independent inspection of HD-tDCS-induced changes in neurophysiology, time-on-task effects, and ongoing task processing. Secondly, as indicated by the results from the present study and prior research (Alnes et al., 2023; Arnett et al., 2022; Lendner et al., 2020), the results might be highly sensitive to the frequency ranges used to extract aperiodic parameters. While the use of two different fits might have been avoided by the use of a “knee” (Gerster et al., 2022) when fitting the data, this yielded highly suboptimal fits to the present data. This highlights that future refinements of parametrization algorithms may aid in more clearly grasping the different spectral components. Third, it must be noted that in order to explore the different hypotheses of the present study, a high number of different models were tested, which increases the likelihood of incurring Type I errors. In line with this prior limitation, it must also be considered that the indirect effects observed in the parallel mediation models were only observed when a directional hypothesis was assumed (i.e., using confidence intervals at 90%) and that some of the observed effect sizes were considerably low, though not negligible. These aspects should be taken into account for a more cautious interpretation of the results. Furthermore, it must be noted that the use of the offset from the higher frequency range might be controversial, as it has not been reported/explored in the above-cited studies that also employ a higher frequency range. Using a smaller and higher frequency range at the same time might increase the correlation between exponent and offset measures, rendering the measures less independent. Finally, regarding stimulation outcomes, whilst the single-blind procedure was successful, subjective beliefs about the stimulation were only recorded after completing the experiment. Recent evidence, however, suggests that attitudes and expectations toward the effects of stimulation (i.e., the belief that stimulation has a positive or negative effect on task performance) could have an effect on task performance beyond the actual effect of stimulation (Rabipour et al., 2018; Villa-Sánchez et al., 2025). This aspect was not controlled for in the current experiment and should be taken into account in future research.

Based on the findings outlined in the present study and their limitations, future research is needed to better grasp the intricate but promising relationship between aperiodic EEG data and tDCS-induced effects in vigilance performance. Given the sparse evidence of aperiodic EEG data in the context of tDCS studies, the present findings require further replication, likely using larger sample sizes. Future studies would also benefit from the use of both rs-EEG in addition to on-task EEG data, to better explore the role of baseline EEG activity, as well as to better understanding the interaction of task-induced and tDCS-induced neurophysiological changes. For the on-task EEG data, a promising approach would be a more specific look into aperiodic data on a trial-by-trial basis (Arnett et al., 2022; Gyurkovics et al., 2022). Lastly, incorporating complementary measures of excitation/inhibition (E/I) balance, such as MRS-derived glutamate/GABA ratios (McKeon et al., 2024), could further refine our mechanistic understanding of cognitive-load-dependent effects of transcranial electrical stimulation (tES). As noted in the introduction, MRS- and EEG-based markers of E/I do not always align (Cohen Kadosh, 2025), underscoring the need for multimodal approaches. This is particularly relevant given that the efficacy of tES likely depends on a complex interplay of intra- and inter-individual neurobiological factors (Li et al., 2015; López-Alonso et al., 2014, 2015). For instance, Zacharopoulos et al. (2025) demonstrated that tRNS yielded behavioral benefits only when specific MRS-based E/I profiles co-occurred with particular resting-state fMRI connectivity patterns. Therefore, future research should aim to integrate these discrepant or interacting effects based on neurophysiological, neurochemical, and network or functional data to more precisely predict and optimize the effects of tES interventions.

### 4.1 Conclusions

This study offers preliminary insights that contribute to our understanding of cognitive-load dependent effects of HD-tDCS on the EV decrement by considering aperiodic parameters as a mediating factor or a potential baseline predictor. The present study underscores the relevance of using adequate or functionally relevant frequency ranges to extract aperiodic parameters, as stimulation-related effects were more evident when using a higher frequency range (30–45 Hz compared to 1–35 Hz). Furthermore, the results highlight the need to consider task-induced neural activity when anticipating the efficacy of tDCS interventions: in a high-load but not overdemanding task, further increasing the E/I balance with HD-tDCS leads to a mitigated EV decrement, potentially by facilitating the firing of near-threshold neurons involved in task-relevant processes. In a low-demand task, further increasing the E/I balance with HD-tDCS leads to a more pronounced EV decrement, potentially due to the facilitation of task-irrelevant processes. In parallel, a push-pull relationship between the aperiodic exponent and offset was observed, countering the effect observed in each task type, and reducing the overall effect. In summary, the study presents novel insights into the potential of aperiodic EEG markers in elucidating the neurophysiological effects by which HD-tDCS can modulate the vigilance decrement. While the findings are encouraging, given that many effects where only observed under directional hypothesis testing, they represent an initial and still exploratory step that warrants replications and extension. Further research will be critical for developing more precise and individualized applications of tDCS for cognitive modulation.

## Data Availability

The data analyzed in this study is subject to the following licenses/restrictions: the datasets analyzed for this study are available on request from the corresponding author [KH]. Requests to access these datasets should be directed to Klara Hemmerich (klara.hemmerich@gmail.com).

## References

[B1] Abrahamyan A. Clifford C. W. G. Arabzadeh E. Harris J. A. (2011). Improving visual sensitivity with subthreshold transcranial magnetic stimulation. J. Neurosci. 31, 3290–3294. doi: 10.1523/JNEUROSCI.6256-10.2011 21368040 PMC6623934

[B2] Ahmad J. Ellis C. Leech R. Voytek B. Garces P. Jones E. . (2022). From mechanisms to markers: novel noninvasive EEG proxy markers of the neural excitation and inhibition system in humans. Transl. Psychiatry 12:467. doi: 10.1038/s41398-022-02218-z 36344497 PMC9640647

[B3] Alam M. Truong D. Q. Khadka N. Bikson M. (2016). Spatial and polarity precision of concentric high-definition transcranial direct current stimulation (HD-tDCS). Phys. Med. Biol. 61, 4506–4521. doi: 10.1088/0031-9155/61/12/4506 27223853

[B4] Alnes S. L. Bächlin L. Z. M. Schindler K. Tzovara A. (2023). Neural complexity and the spectral slope characterise auditory processing in wakefulness and sleep. Euro. J. Neurosci. 59, 822–841. doi: 10.22541/au.167850558.84503257/v1 38100263

[B5] Antal A. Luber B. Brem A.-K. Bikson M. Brunoni A. R. Cohen Kadosh R. . (2022). Non-invasive brain stimulation and neuroenhancement. Clin. Neurophysiol. Pract. 7, 146–165. doi: 10.1016/j.cnp.2022.05.002 35734582 PMC9207555

[B6] Arnett A. B. Peisch V. Levin A. R. (2022). The role of aperiodic spectral slope in event-related potentials and cognition among children with and without attention deficit hyperactivity disorder. J. Neurophysiol. 128, 1546–1554. doi: 10.1152/jn.00295.2022 36382902 PMC9902214

[B7] Barger L. K. Ayas N. T. Cade B. E. Cronin J. W. Rosner B. Speizer F. E. . (2006). Impact of extended-duration shifts on medical errors, adverse events, and attentional failures. PLoS Med. 3:e487. doi: 10.1371/journal.pmed.0030487 17194188 PMC1705824

[B8] Bates D. Mächler M. Bolker B. Walker S. (2015). Fitting linear mixed-effects models using lme4. J. Stat. Softw. 67, 1–48. doi: 10.18637/jss.v067.i01

[B9] Benwell C. S. Y. Learmonth G. Miniussi C. Harvey M. Thut G. (2015). Non-linear effects of transcranial direct current stimulation as a function of individual baseline performance: evidence from biparietal tDCS influence on lateralized attention bias. Cortex 69, 152–165. doi: 10.1016/j.cortex.2015.05.007 26073146

[B10] Bergmann T. O. Hartwigsen G. (2021). Inferring causality from noninvasive brain stimulation in cognitive neuroscience. J. Cogn. Neurosci. 33, 195–225. doi: 10.1162/jocn_a_01591 32530381

[B11] Bestmann S. De Berker A. O. Bonaiuto J. (2015). Understanding the behavioural consequences of noninvasive brain stimulation. Trends Cogn. Sci. 19, 13–20. doi: 10.1016/j.tics.2014.10.003 25467129

[B12] Bikson M. name A. Rahman A. (2013). Origins of specificity during tDCS: anatomical, activity-selective, and input-bias mechanisms. Front. Hum. Neurosci. 7:688. doi: 10.3389/fnhum.2013.00688 24155708 PMC3800813

[B13] Boksem M. A. S. Meijman T. F. Lorist M. M. (2005). Effects of mental fatigue on attention: an ERP study. Cogn. Brain Res. 25, 107–116. doi: 10.1016/j.cogbrainres.2005.04.011 15913965

[B14] Brake N. Duc F. Rokos A. Arseneau F. Shahiri S. Khadra A. . (2024). A neurophysiological basis for aperiodic EEG and the background spectral trend. Nat. Commun. 15:1514. doi: 10.1038/s41467-024-45922-8 38374047 PMC10876973

[B15] Braunsmann L. Beermann F. Strüder H. K. Abeln V. (2024). Self-selected versus imposed running intensity and the acute effects on mood, cognition, and (a)periodic brain activity. Cogn. Neurodyn. 18, 2221–2241. doi: 10.1007/s11571-024-10084-2 39555283 PMC11564500

[B16] Brosnan M. B. Arvaneh M. Harty S. Maguire T. O'Connell R. Robertson I. H. . (2018). Prefrontal modulation of visual processing and sustained attention in aging, a tDCS–EEG coregistration approach. J. Cogn. Neurosci. 30, 1630–1645. doi: 10.1162/jocn_a_01307 30004847

[B17] Bunai T. Hirosawa T. Kikuchi M. Fukai M. Yokokura M. Ito S. . (2021). tDCS-induced modulation of GABA concentration and dopamine release in the human brain: a combination study of magnetic resonance spectroscopy and positron emission tomography. Brain Stimul. 14, 154–160. doi: 10.1016/j.brs.2020.12.010 33359603

[B18] Buzsáki G. Anastassiou C. A. Koch C. (2012). The origin of extracellular fields and currents—EEG, ECoG, LFP and spikes. Nat. Rev. Neurosci. 13, 407–420. doi: 10.1038/nrn3241 22595786 PMC4907333

[B19] Catroppa C. Anderson V. (2005). A prospective study of the recovery of attention from acute to 2 years following pediatric traumatic brain injury. J. Int. Neuropsychol. Soc. 11, 84–98. doi: 10.1017/S1355617705050101 15686611

[B20] Clayton M. S. Yeung N. Cohen Kadosh R. (2015). The roles of cortical oscillations in sustained attention. Trends Cogn. Sci. 19, 188–195. doi: 10.1016/j.tics.2015.02.004 25765608

[B21] Cohen Kadosh R. (2025). Rethinking excitation/inhibition balance in the human brain. Nat. Rev. Neurosci. 26, 451–452. doi: 10.1038/s41583-025-00943-0 40551004

[B22] Coll-Martín T. Román-Caballero R. Martínez-Caballero M. D. R. Martín-Sánchez P. D. C. Trujillo L. Cásedas L. . (2023). The ANTI-Vea-UGR platform: a free online resource to measure attentional networks (alertness, orienting, and executive control) functioning and executive/arousal vigilance. J. Intell. 11:181. doi: 10.3390/jintelligence11090181 37754910 PMC10532513

[B23] Coulborn S. Bowman H. Miall R. C. Fernández-Espejo D. (2020). Effect of tDCS over the right inferior parietal lobule on mind-wandering propensity. Front. Hum. Neurosci. 14:230. doi: 10.3389/fnhum.2020.00230 32655387 PMC7325883

[B24] Craig A. Tran Y. Wijesuriya N. Nguyen H. (2012). Regional brain wave activity changes associated with fatigue: regional brain wave activity and fatigue. Psychophysiology 49, 574–582. doi: 10.1111/j.1469-8986.2011.01329.x 22324302

[B25] Dakwar-Kawar O. Mairon N. Hochman S. Berger I. Cohen Kadosh R. Nahum M. (2023). Transcranial random noise stimulation combined with cognitive training for treating ADHD: a randomized, sham-controlled clinical trial. Transl. Psychiatry 13:271. doi: 10.1038/s41398-023-02547-7 37528107 PMC10394047

[B26] Dakwar-Kawar O. Mentch-Lifshits T. Hochman S. Mairon N. Cohen R. Balasubramani P. . (2024). Aperiodic and periodic components of oscillatory brain activity in relation to cognition and symptoms in pediatric ADHD. Cereb. Cortex 34:bhae236. doi: 10.1093/cercor/bhae236 38858839

[B27] Delorme A. Makeig S. (2004). EEGLAB: an open source toolbox for analysis of single-trial EEG dynamics including independent component analysis. J. Neurosci. Methods 134, 9–21. doi: 10.1016/j.jneumeth.2003.10.009 15102499

[B28] Dockery C. A. Hueckel-Weng R. Birbaumer N. Plewnia C. (2009). Enhancement of planning ability by transcranial direct current stimulation. J. Neurosci. 29, 7271–7277. doi: 10.1523/JNEUROSCI.0065-09.2009 19494149 PMC6666475

[B29] Donoghue T. (2025). A systematic review of aperiodic neural activity in clinical investigations. Eur. J. Neurosci. 62, 1–30. doi: 10.1111/ejn.70255 41074683 PMC12514750

[B30] Donoghue T. Dominguez J. Voytek B. (2020a). Electrophysiological frequency band ratio measures conflate periodic and aperiodic neural activity. Eneuro 7:ENEURO.0192-20.2020. doi: 10.1523/ENEURO.0192-20.2020 32978216 PMC7768281

[B31] Donoghue T. Haller M. Peterson E. J. Varma P. Sebastian P. Gao R. . (2020b). Parameterizing neural power spectra into periodic and aperiodic components. Nat. Neurosci. 23, 1655–1665. doi: 10.1038/s41593-020-00744-x 33230329 PMC8106550

[B32] Donoghue T. Schaworonkow N. Voytek B. (2021). Methodological considerations for studying neural oscillations. Euro. J. Neurosci. 55, 3502–3527. doi: 10.31234/osf.io/hvd67 34268825 PMC8761223

[B33] Duncan N. W. Wiebking C. Northoff G. (2014). Associations of regional GABA and glutamate with intrinsic and extrinsic neural activity in humans—a review of multimodal imaging studies. Neurosci. Biobehav. Rev. 47, 36–52. doi: 10.1016/j.neubiorev.2014.07.016 25066091

[B34] Dyke K. Pépés S. E. Chen C. Kim S. Sigurdsson H. P. Draper A. . (2017). Comparing GABA-dependent physiological measures of inhibition with proton magnetic resonance spectroscopy measurement of GABA using ultra-high-field MRI. NeuroImage 152, 360–370. doi: 10.1016/j.neuroimage.2017.03.011 28284797 PMC5440178

[B35] Edwards D. Cortes M. Datta A. Minhas P. Wassermann E. M. Bikson M. (2013). Physiological and modeling evidence for focal transcranial electrical brain stimulation in humans: a basis for high-definition tDCS. NeuroImage 74, 266–275. doi: 10.1016/j.neuroimage.2013.01.042 23370061 PMC4359173

[B36] Fassi L. Cohen Kadosh R. (2021). Letter to the editor: how some brain stimulation studies fail to evaluate blinding adequately. J. Psychiatr. Res. 137, 452–453. doi: 10.1016/j.jpsychires.2021.03.020 33798970

[B37] Fassi L. Hochman S. Daskalakis Z. J. Blumberger D. M. Kadosh R. C. (2023). The importance of individual beliefs in assessing treatment efficacy: insights from neurostimulation studies. eLife 12:RP88889. doi: 10.7554/eLife.88889.3 PMC10977967 38547008

[B38] Fertonani A. Ferrari C. Miniussi C. (2015). What do you feel if I apply transcranial electric stimulation? Safety, sensations and secondary induced effects. Clin. Neurophysiol. 126, 2181–2188. doi: 10.1016/j.clinph.2015.03.015 25922128

[B39] Fertonani A. Miniussi C. (2017). Transcranial electrical stimulation: what we know and do not know about mechanisms. Neuroscientist 23, 109–123. doi: 10.1177/1073858416631966 26873962 PMC5405830

[B40] Filmer H. L. Ehrhardt S. E. Bollmann S. Mattingley J. B. Dux P. E. (2019). Accounting for individual differences in the response to tDCS with baseline levels of neurochemical excitability. Cortex 115, 324–334. doi: 10.1016/j.cortex.2019.02.012 30903834

[B41] Gan T. Huang Y. Hao X. Hu L. Zheng Y. Yang Z. (2022). Anodal tDCS over the left frontal eye field improves sustained visual search performance. Perception 51, 263–275. doi: 10.1177/03010066221086446 35275023

[B42] Gao R. Peterson E. J. Voytek B. (2017). Inferring synaptic excitation/inhibition balance from field potentials. NeuroImage 158, 70–78. doi: 10.1016/j.neuroimage.2017.06.078 28676297

[B43] Gao Y. Koyun A. H. Roessner V. Stock A.-K. Mückschel M. Colzato L. . (2025). Transcranial direct current stimulation and methylphenidate interact to increase cognitive persistence as a core component of metacontrol: evidence from aperiodic activity analyses. Brain Stimul. 18, 720–729. doi: 10.1016/j.brs.2025.03.024 40180219

[B44] Gerster M. Waterstraat G. Litvak V. Lehnertz K. Schnitzler A. Florin E. . (2022). Separating neural oscillations from aperiodic 1/f activity: challenges and recommendations. Neuroinformatics. 20, 991–1012. doi: 10.1007/s12021-022-09581-8 35389160 PMC9588478

[B45] Gilden D. L. (2001). Cognitive emissions of 1/f noise. Psychol. Rev. 108, 33–56. doi: 10.1037/0033-295X.108.1.33 11212631

[B46] Gyurkovics M. Clements G. M. Low K. A. Fabiani M. Gratton G. (2022). Stimulus-induced changes in 1/f-like background activity in EEG. J. Neurosci. 42, 7144–7151. doi: 10.1523/JNEUROSCI.0414-22.2022 35970561 PMC9480870

[B47] Harty S. Sella F. Cohen Kadosh R. (2017). Mind the brain: the mediating and moderating role of neurophysiology. Trends Cogn. Sci. 21, 2–5. doi: 10.1016/j.tics.2016.11.002 27931847

[B48] Hayes A. F. (2022). Introduction to Mediation, Moderation, and Conditional Process Analysis. A Regression-Based Approach, 3rd Edn. New York, NY: The Guilford Press.

[B49] He B. J. (2014). Scale-free brain activity: past, present, and future. Trends Cogn. Sci. 18, 480–487. doi: 10.1016/j.tics.2014.04.003 24788139 PMC4149861

[B50] He B. J. Zempel J. M. Snyder A. Z. Raichle M. E. (2010). The temporal structures and functional significance of scale-free brain activity. Neuron 66, 353–369. doi: 10.1016/j.neuron.2010.04.020 20471349 PMC2878725

[B51] Hemmerich K. Luna F. G. Martín-Arévalo E. Lupiáñez J. (2025). Understanding vigilance and its decrement: theoretical, contextual, and neural insights. Front. Cogn. 4:1617561. doi: 10.3389/fcogn.2025.1617561 PMC13271136 42339255

[B52] Hemmerich K. Lupiáñez J. Luna F. G. Martín-Arévalo E. (2023). The mitigation of the executive vigilance decrement via HD-tDCS over the right posterior parietal cortex and its association with neural oscillations. Cereb. Cortex 33, 6761–6771. doi: 10.1093/cercor/bhac540 36646467

[B53] Hemmerich K. Lupiáñez J. Martín-Arévalo E. (2024). HD-tDCS mitigates the executive vigilance decrement only under high cognitive demands. Sci. Rep. 14:7865. doi: 10.1038/s41598-024-57917-y 38570619 PMC10991279

[B54] Ho K.-A. Taylor J. L. Loo C. K. (2015). Comparison of the effects of transcranial random noise stimulation and transcranial direct current stimulation on motor cortical excitability. J. ECT 31, 67–72. doi: 10.1097/YCT.0000000000000155 25010032

[B55] Höhn C. Hahn M. A. Lendner J. D. Hoedlmoser K. (2024). Spectral slope and Lempel–Ziv complexity as robust markers of brain states during sleep and wakefulness. Eneuro 11, 1–17. doi: 10.1523/ENEURO.0259-23.2024 38471778 PMC10978822

[B56] Immink M. A. Cross Z. R. Chatburn A. Baumeister J. Schlesewsky M. Bornkessel-Schlesewsky I. (2021). Resting-state aperiodic neural dynamics predict individual differences in visuomotor performance and learning. Hum. Mov. Sci. 78:102829. doi: 10.1016/j.humov.2021.102829 34139391

[B57] Inukai Y. Saito K. Sasaki R. Tsuiki S. Miyaguchi S. Kojima S. . (2016). Comparison of three non-invasive transcranial electrical stimulation methods for increasing cortical excitability. Front. Hum. Neurosci. 10:668. doi: 10.3389/fnhum.2016.00668 28082887 PMC5186778

[B58] Kang J. Wu J. Huang X. Mao W. Li X. (2025). Differential effects of left DLPFC anodal and cathodal tDCS interventions on the brain in children with autism: a randomized controlled trial. IBRO Neurosci. Rep. 18, 171–179. doi: 10.1016/j.ibneur.2025.01.005 39896718 PMC11787616

[B59] Kasten F. H. Lattmann R. Strüber D. Herrmann C. S. (2024). Decomposing the effects of α-tACS on brain oscillations and aperiodic 1/f activity. Brain Stimul. 17, 721–723. doi: 10.1016/j.brs.2024.05.015 38823439

[B60] Kharoufah H. Murray J. Baxter G. Wild G. (2018). A review of human factors causations in commercial air transport accidents and incidents: from to 2000–2016. Progress Aerosp. Sci. 99, 1–13. doi: 10.1016/j.paerosci.2018.03.002

[B61] Krause B. Márquez-Ruiz J. Kadosh R. C. (2013). The effect of transcranial direct current stimulation: a role for cortical excitation/inhibition balance? Front. Hum. Neurosci. 7:602. doi: 10.3389/fnhum.2013.00602 24068995 PMC3781319

[B62] Krüger J. K. Suchan B. (2015). Humans are still the critical factor in aviation security. Aerosp. Med. Hum. Perform. 86, 915–917. doi: 10.3357/AMHP.4315.2015 26564681

[B63] Kuo M.-F. Nitsche M. A. (2012). Effects of transcranial electrical stimulation on cognition. Clin. EEG Neurosci. 43, 192–199. doi: 10.1177/1550059412444975 22956647

[B64] Lee J.-M. Kim P.-J. Kim H.-G. Hyun H.-K. Kim Y. J. Kim J.-W. . (2020). Analysis of brain connectivity during nitrous oxide sedation using graph theory. Sci. Rep. 10:2354. doi: 10.1038/s41598-020-59264-0 32047246 PMC7012909

[B65] Lee M. D. Wagenmakers E.-J. (2014). Bayesian Cognitive Modeling: A Practical Course. Cambridge: Cambridge University Press. doi: 10.1017/CBO9781139087759

[B66] Lendner J. D. Helfrich R. F. Mander B. A. Romundstad L. Lin J. J. Walker M. P. . (2020). An electrophysiological marker of arousal level in humans. eLife 9:e55092. doi: 10.7554/eLife.55092.sa2 32720644 PMC7394547

[B67] Lendner J. D. Niethard N. Mander B. A. Van Schalkwijk F. J. Schuh-Hofer S. Schmidt H. . (2023). Human REM sleep recalibrates neural activity in support of memory formation. Sci. Adv. 9:eadj1895. doi: 10.1126/sciadv.adj1895 37624898 PMC10456851

[B68] Li L. M. Uehara K. Hanakawa T. (2015). The contribution of interindividual factors to variability of response in transcranial direct current stimulation studies. Front. Cell. Neurosci. 9:181. doi: 10.3389/fncel.2015.00181 26029052 PMC4428123

[B69] Looi C. Y. Duta M. Brem A.-K. Huber S. Nuerk H.-C. Cohen Kadosh R. (2016). Combining brain stimulation and video game to promote long-term transfer of learning and cognitive enhancement. Sci. Rep. 6:22003. doi: 10.1038/srep22003 26902664 PMC4763231

[B70] López-Alonso V. Cheeran B. Río-Rodríguez D. Fernández-del-Olmo M. (2014). Inter-individual variability in response to non-invasive brain stimulation paradigms. Brain Stimul. 7, 372–380. doi: 10.1016/j.brs.2014.02.004 24630849

[B71] López-Alonso V. Fernández-del-Olmo M. Costantini A. Gonzalez-Henriquez J. J. Cheeran B. (2015). Intra-individual variability in the response to anodal transcranial direct current stimulation. Clin. Neurophysiol. 126, 2342–2347. doi: 10.1016/j.clinph.2015.03.022 25922127

[B72] Luna F. G. Barttfeld P. Martín-Arévalo E. Lupiáñez J. (2021). The ANTI-Vea task: analyzing the executive and arousal vigilance decrements while measuring the three attentional networks. Psicol. J. 42, 1–26. doi: 10.2478/psicolj-2021-0001

[B73] Luna F. G. Barttfeld P. Martín-Arévalo E. Lupiáñez J. (2022). Cognitive load mitigates the executive but not the arousal vigilance decrement. Conscious. Cogn. 98:103263. doi: 10.1016/j.concog.2021.103263 34954544

[B74] Luna F. G. Marino J. Roca J. Lupiáñez J. (2018). Executive and arousal vigilance decrement in the context of the attentional networks: the ANTI-Vea task. J. Neurosci. Methods 306, 77–87. doi: 10.1016/j.jneumeth.2018.05.011 29791865

[B75] Luna F. G. Román-Caballero R. Barttfeld P. Lupiáñez J. Martín-Arévalo E. (2020). A high-definition tDCS and EEG study on attention and vigilance: brain stimulation mitigates the executive but not the arousal vigilance decrement. Neuropsychologia 142:107447. doi: 10.1016/j.neuropsychologia.2020.107447 32243885

[B76] MacKinnon D. P. Krull J. L. Lockwood C. M. (2000). Equivalence of the mediation, confounding and suppression effect. Prev. Sci. 1, 173–181. doi: 10.1023/A:1026595011371 11523746 PMC2819361

[B77] MacKinnon D. P. Lockwood C. M. Hoffman J. M. West S. G. (2010). A comparison of methods to test mediation and other intervening variable effects. Psychol. Methods 7:83. 11928892 10.1037/1082-989x.7.1.83 PMC2819363

[B78] Mackworth N. H. (1948). The breakdown of vigilance during prolonged visual search. Q. J. Exp. Psychol. 1, 6–21. doi: 10.1080/17470214808416738

[B79] Manning J. R. Jacobs J. Fried I. Kahana M. J. (2009). Broadband shifts in local field potential power spectra are correlated with single-neuron spiking in humans. J. Neurosci. 29, 13613–13620. doi: 10.1523/JNEUROSCI.2041-09.2009 19864573 PMC3001247

[B80] Martínez-Pérez V. Andreu A. Sandoval-Lentisco A. Tortajada M. Palmero L. B. Castillo A. . (2023). Vigilance decrement and mind-wandering in sustained attention tasks: two sides of the same coin? Front. Neurosci. 17:1122406. doi: 10.3389/fnins.2023.1122406 37056308 PMC10086236

[B81] Masina F. Napoli E. Santacesaria P. Giustiniani A. Zago S. Marino M. . (2025). Transcranial alternating current stimulation selectively modulates aperiodic EEG component: unveiling alternative mechanisms of modulation. Clin. Neurophysiol. 177:2110929. doi: 10.1016/j.clinph.2025.2110929 40652841

[B82] McKeon S. D. Perica M. I. Parr A. C. Calabro F. J. Foran W. Hetherington H. . (2024). Aperiodic EEG and 7T MRSI evidence for maturation of E/I balance supporting the development of working memory through adolescence. Dev. Cogn. Neurosci. 66:101373. doi: 10.1016/j.dcn.2024.101373 38574406 PMC11000172

[B83] Medel V. Irani M. Crossley N. Ossandón T. Boncompte G. (2023). Complexity and 1/f slope jointly reflect brain states. Sci. Rep. 13:21700. doi: 10.1038/s41598-023-47316-0 38065976 PMC10709649

[B84] Miniussi C. Harris J. A. Ruzzoli M. (2013). Modelling non-invasive brain stimulation in cognitive neuroscience. Neurosci. Biobehav. Rev. 37, 1702–1712. doi: 10.1016/j.neubiorev.2013.06.014 23827785

[B85] Muthukumaraswamy S. D. (2013). High-frequency brain activity and muscle artifacts in MEG/EEG: a review and recommendations. Front. Hum. Neurosci. 7:138. doi: 10.3389/fnhum.2013.00138 23596409 PMC3625857

[B86] Nelson J. T. McKinley R. A. Golob E. J. Warm J. S. Parasuraman R. (2014). Enhancing vigilance in operators with prefrontal cortex transcranial direct current stimulation (tDCS). NeuroImage 85, 909–917. doi: 10.1016/j.neuroimage.2012.11.061 23235272

[B87] Ouyang G. Hildebrandt A. Schmitz F. Herrmann C. S. (2020). Decomposing alpha and 1/f brain activities reveals their differential associations with cognitive processing speed. NeuroImage 205:116304. doi: 10.1016/j.neuroimage.2019.116304 31654760

[B88] Pathania A. Schreiber M. Miller M. W. Euler M. J. Lohse K. R. (2021). Exploring the reliability and sensitivity of the EEG power spectrum as a biomarker. Int. J. Psychophysiol. 160, 18–27. doi: 10.1016/j.ijpsycho.2020.12.002 33340559

[B89] Pei L. Northoff G. Ouyang G. (2023). Comparative analysis of multifaceted neural effects associated with varying endogenous cognitive load. Commun. Biol. 6:795. doi: 10.1038/s42003-023-05168-4 37524883 PMC10390511

[B90] Pershin I. Candrian G. Münger M. Baschera G.-M. Rostami M. Eich D. . (2023). Vigilance described by the time-on-task effect in EEG activity during a cued Go/NoGo task. Int. J. Psychophysiol. 183, 92–102. doi: 10.1016/j.ijpsycho.2022.11.015 36455720

[B91] Pertermann M. Mückschel M. Adelhöfer N. Ziemssen T. Beste C. (2019). On the interrelation of 1/f neural noise and norepinephrine system activity during motor response inhibition. J. Neurophysiol. 121, 1633–1643. doi: 10.1152/jn.00701.2018 30811254

[B92] Pi Y. Yan J. Pscherer C. Gao S. Mückschel M. Colzato L. . (2024). Interindividual aperiodic resting-state EEG activity predicts cognitive-control styles. Psychophysiology 61:e14576. doi: 10.1111/psyp.14576 38556626

[B93] Pievsky M. A. McGrath R. E. (2018). The neurocognitive profile of attention-deficit/hyperactivity disorder: a review of meta-analyses. Arch. Clin. Neuropsychol. 33, 143–157. doi: 10.1093/arclin/acx055 29106438

[B94] Podvalny E. Noy N. Harel M. Bickel S. Chechik G. Schroeder C. E. . (2015). A unifying principle underlying the extracellular field potential spectral responses in the human cortex. J. Neurophysiol. 114, 505–519. doi: 10.1152/jn.00943.2014 25855698 PMC4509389

[B95] Poil S.-S. Hardstone R. Mansvelder H. D. Linkenkaer-Hansen K. (2012). Critical-state dynamics of avalanches and oscillations jointly emerge from balanced excitation/inhibition in neuronal networks. J. Neurosci. 32, 9817–9823. doi: 10.1523/JNEUROSCI.5990-11.2012 22815496 PMC3553543

[B96] Rabipour S. Wu A. D. Davidson P. S. R. Iacoboni M. (2018). Expectations may influence the effects of transcranial direct current stimulation. Neuropsychologia 119, 524–534. doi: 10.1016/j.neuropsychologia.2018.09.005 30227147

[B97] Rico-Picó J. Moyano S. Conejero Á. Hoyo Á. Ballesteros-Duperón M. Á. Rueda M. R. (2023). Early development of electrophysiological activity: contribution of periodic and aperiodic components of the EEG signal. Psychophysiology 60:e14360. doi: 10.1111/psyp.14360 37322838

[B98] Robertson M. M. Furlong S. Voytek B. Donoghue T. Boettiger C. A. Sheridan M. A. (2019). EEG power spectral slope differs by ADHD status and stimulant medication exposure in early childhood. J. Neurophysiol. 122, 2427–2437. doi: 10.1152/jn.00388.2019 31619109 PMC6966317

[B99] Roe J. M. Nesheim M. Mathiesen N. C. Moberget T. Alnæs D. Sneve M. H. (2016). The effects of tDCS upon sustained visual attention are dependent on cognitive load. Neuropsychologia 80, 1–8. doi: 10.1016/j.neuropsychologia.2015.11.005 26556389

[B100] Rossi S. Hallett M. Rossini P. M. Pascual-Leone A. (2009). Safety, ethical considerations, and application guidelines for the use of transcranial magnetic stimulation in clinical practice and research. Clin. Neurophysiol. 120, 2008–2039. doi: 10.1016/j.clinph.2009.08.016 19833552 PMC3260536

[B101] Rossini P. M. Burke D. Chen R. Cohen L. G. Daskalakis Z. Di Iorio R. . (2015). Non-invasive electrical and magnetic stimulation of the brain, spinal cord, roots and peripheral nerves: basic principles and procedures for routine clinical and research application. An updated report from an I.F.C.N. Committee. Clin. Neurophysiol. 126, 1071–1107. doi: 10.1016/j.clinph.2015.02.001 25797650 PMC6350257

[B102] Roy L. B. Sparing R. Fink G. R. Hesse M. D. (2015). Modulation of attention functions by anodal tDCS on right PPC. Neuropsychologia 74, 96–107. doi: 10.1016/j.neuropsychologia.2015.02.028 25721567

[B103] Sheffield J. G. Raz G. Sella F. Kadosh R. C. (2020). How can noise alter neurophysiology in order to improve human behaviour? A combined tRNS and EEG study [Preprint]. bioRxiv. doi: 10.1101/2020.01.09.900118

[B104] Shrout P. E. Bolger N. (2002). Mediation in experimental and nonexperimental studies: new procedures and recommendations. Psychol. Methods 7, 422–445. doi: 10.1037/1082-989X.7.4.422 12530702

[B105] Simula S. Makhalova J. Pizzo F. Garnier E. Damiani G. Mercadal B. . (2024). Impact of transcranial electrical stimulation on simultaneous stereoelectroencephalography recordings: a randomized sham-controlled study. Clin. Neurophysiol. 166, 211–222. doi: 10.1016/j.clinph.2024.08.003 39182340

[B106] Stagg C. J. Bestmann S. Constantinescu A. O. Moreno Moreno L. Allman C. Mekle R. . (2011). Relationship between physiological measures of excitability and levels of glutamate and GABA in the human motor cortex. J. Physiol. 589, 5845–5855. doi: 10.1113/jphysiol.2011.216978 22005678 PMC3249054

[B107] Thomson D. R. Besner D. Smilek D. (2015). A resource-control account of sustained attention: evidence from mind-wandering and vigilance paradigms. Perspect. Psychol. Sci. 10, 82–96. doi: 10.1177/1745691614556681 25910383

[B108] Turri C. Di Dona G. Santoni A. Zamfira D. A. Franchin L. Melcher D. . (2023). Periodic and aperiodic EEG features as potential markers of developmental dyslexia. Biomedicines 11:1607. doi: 10.3390/biomedicines11061607 37371702 PMC10296084

[B109] van Bueren N. E. R. van Der Ven S. H. G. Hochman S. Sella F. Cohen Kadosh R. (2023). Human neuronal excitation/inhibition balance explains and predicts neurostimulation induced learning benefits. PLoS Biol. 21:e3002193. doi: 10.1371/journal.pbio.3002193 37651315 PMC10470965

[B110] Venugopal R. Sasidharan A. Bhowmick K. Nagaraj N. Udupa K. John J. P. . (2025). Personalized theta transcranial alternating current stimulation and gamma transcranial alternating current stimulation bring differential neuromodulatory effects on the resting electroencephalogram: characterizing the temporal, spatial, and spectral dimensions of transcranial alternating current stimulation. Neuromodul. Technol. Neural Interface 28, 425–433. doi: 10.1016/j.neurom.2024.08.008 39425734

[B111] Villa-Sánchez B. Hooyman A. Schaefer S. Y. (2025). The influence of informational priming on motor expectancy in transcranial direct current stimulation (tDCS). Exp. Brain Res. 243:157. doi: 10.1007/s00221-025-07110-y 40439933

[B112] Voytek B. Knight R. T. (2015). Dynamic network communication as a unifying neural basis for cognition, development, aging, and disease. Biol. Psychiatry 77, 1089–1097. doi: 10.1016/j.biopsych.2015.04.016 26005114 PMC4443259

[B113] Vural G. Soldini A. Padberg F. Karsli B. Zinchenko A. Goerigk S. . (2024). Exploring the effects of prefrontal transcranial direct current stimulation on brain metabolites: a concurrent tDCS - MRS study. Hum. Brain Mapp. 45:e70097. doi: 10.1002/hbm.70097 39688161 PMC11651192

[B114] Warm J. S. Parasuraman R. Matthews G. (2008). Vigilance requires hard mental work and is stressful. Hum. Fact. 50, 433–441. doi: 10.1518/001872008X312152 18689050

[B115] Waschke L. Donoghue T. Fiedler L. Smith S. Garrett D. D. Voytek B. . (2021). Modality-specific tracking of attention and sensory statistics in the human electrophysiological spectral exponent. eLife 10:e70068. doi: 10.7554/eLife.70068.sa2 34672259 PMC8585481

[B116] Weber J. Klein T. Abeln V. (2020). Shifts in broadband power and alpha peak frequency observed during long-term isolation. Sci. Rep. 10:17987. doi: 10.1038/s41598-020-75127-0 33093553 PMC7581825

[B117] Wundersitz L. (2019). Driver distraction and inattention in fatal and injury crashes: findings from in-depth road crash data. Traffic Inj. Prev. 20, 696–701. doi: 10.1080/15389588.2019.1644627 31408358

[B118] Zacharopoulos G. Dehghani M. Krause-Sorio B. Near J. Cohen Kadosh R. (2025). Functional connectivity and GABAergic signaling modulate the enhancement effect of neurostimulation on mathematical learning. PLoS Biol. 23:e3003200. doi: 10.1371/journal.pbio.3003200 40591518 PMC12212564

[B119] Zhang C. Stock A.-K. Mückschel M. Hommel B. Beste C. (2023). Aperiodic neural activity reflects metacontrol. Cereb. Cortex 33, 7941–7951. doi: 10.1093/cercor/bhad089 36928696

[B120] Žiburkus J. Cressman J. R. Schiff S. J. (2013). Seizures as imbalanced up states: excitatory and inhibitory conductances during seizure-like events. J. Neurophysiol. 109, 1296–1306. doi: 10.1152/jn.00232.2012 23221405 PMC3602838

