# Correction: Cognitive load-dependent effects of HD-tDCS on the executive vigilance decrement: insights from aperiodic EEG activity

**DOI:** 10.3389/fcogn.2026.1813340

**Published:** 2026-03-04

**Authors:** Klara Hemmerich, Juan Lupiáñez, Elisa Martín-Arévalo, Roi Cohen Kadosh

**Affiliations:** 1 Department of Psychology and Cognitive Science, University of Trento, Rovereto, Italy; 2 Department of Experimental Psychology and Mind, Brain, and Behavior Research Center (CIMCYC), Universidad de Granada, Granada, Spain; 3 School of Psychology, University of Surrey, Guildford, United Kingdom

**Keywords:** aperiodic exponent, aperiodic offset, EEG, executive vigilance, HD-tDCS, spectral parameterization, vigilance

In the published article, there was a mistake in the **Funding** statement. The funding statement for “project PID2024-157672NB-I00” was displayed as “MCIN/AEI/10.13039/501100011033” but should have been displayed as “MICIU/AEI/10.13039/501100011033/FEDER, UE”. The correct statement is “This work was supported by the research project PID2024-157672NB-I00 to EM-A, funded by MICIU/AEI/10.13039/501100011033/FEDER, UE.”

The original version of this article has been updated.

