# Supplementary material for: Cognitive load-dependent effects of HD-tDCS on the executive vigilance decrement: insights from aperiodic EEG activity
Source: Front Cognit. 2026 Jan 22;4:1677285. doi: 10.3389/fcogn.2025.1677285 (PMC13281218; doi:10.3389/fcogn.2025.1677285)
Supplement: Supplementary file 1 [file Supplementary_file_1.docx]

Supplementary Material

# Extraction of alpha peaks after spectral parametrization

For the periodic data (i.e., peaks above the aperiodic exponent), we extracted the centre frequency (CF), power (PW), and bandwidth (BW) for each peak. As can be seen in **Supplementary** **Fig. 1.A**, in the periodic data extracted from spectra in the 1-35 Hz range, the most common CF from the detected peaks was around 10 Hz, i.e., in the alpha range (7.5-12.5 Hz). To ensure consistency in our analysis, we selected the peak with the highest power value from each electrode and recording period for every participant. This way, a single peak could be used to calculate the post-pre change measures. This approach, as depicted in **Supplementary Fig. 1.B**, reduced the number of peaks uniformly across the alpha band, i.e., preserving the distribution of the original data. Consequently, the total number of observations for alpha power was reduced from 2203 to 1793, retaining 81.39% of the data for further analyses. On the other hand, as can be seen in **Supplementary Fig. 1.C**, in the periodic data extracted from the 30-45 Hz spectra, the most common CF was around 36-39 Hz. However, in the gamma range, many electrodes registered no periodic activity (i.e., peaks above the exponent) at all, yielding an insufficient number of observations for further analyses (all peaks constituted 819, from which 721 remained after leaving only the peak with the highest power for each participant’s electrode and recording period).


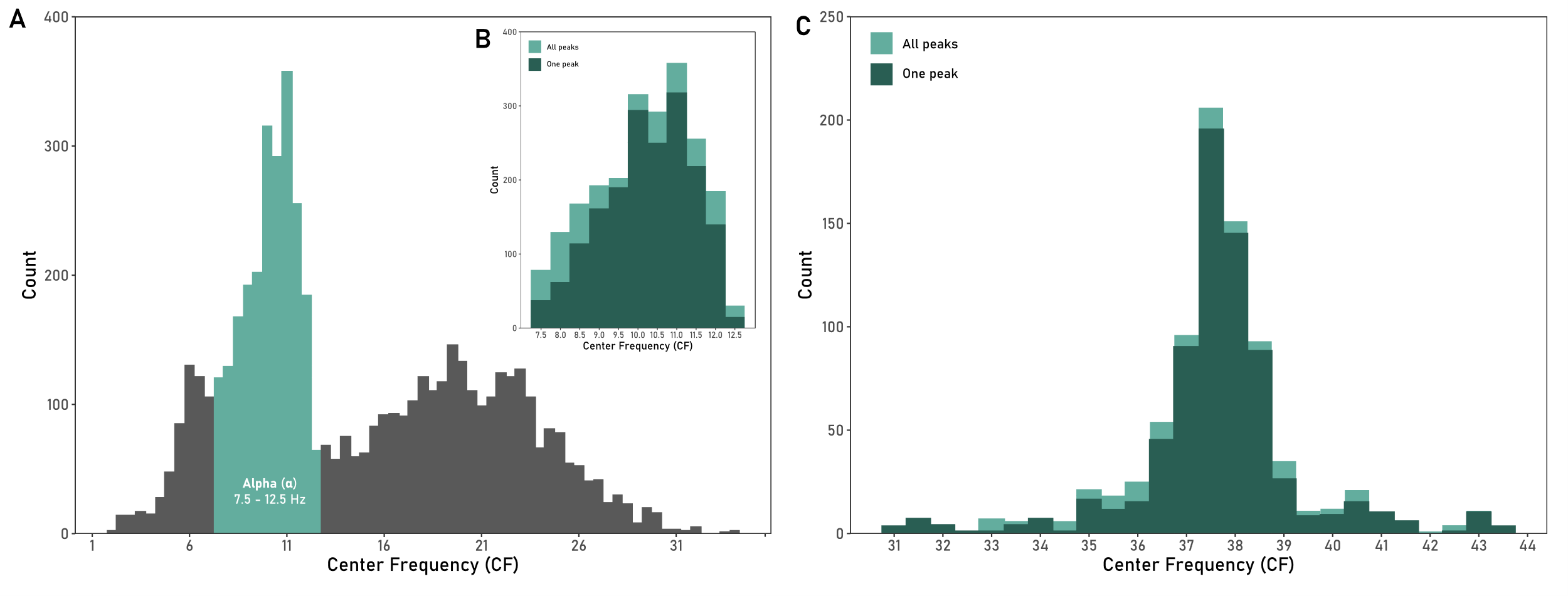


**Suppl. Fig. 1**. (A) Distribution of Centre Frequencies (CF) at a frequency resolution of 0.5 Hz across all datasets, which depicts at which frequency peaks are most common. (B) Distribution of CF within the alpha band (7.5-12.5 Hz) considering all detected peaks (light green) and after reducing the peaks within this narrow-band range to maximum one peak per participant (dark green). (C) Distribution of CF within the gamma band (30-45 Hz) considering all detected peaks (light green) and after reducing the peaks within this narrow-band range to maximum one peak per participant (dark green).

# Blinding efficacy

As this subjective sensation data did not follow a normal distribution (*p* < .001 in all conditions), we performed the Mann-Whitney U test (*N* = 180) to test for the blinding efficacy, completed by a Bayesian independent sample test showing that there is only anecdotal evidence for group-differences for *total discomfort*, *pinching,* and *burning*.

| **Supplementary Table 1**. Sensations between the anodal and sham HD-tDCS groups | | | | |
| --- | --- | --- | --- | --- |
| **Sensation** | **U** | **p** |  | **BF_10_** |
| **Total discomfort** | **4895.00** | **.014** |  | 2.03 |
| Itching | 4550.00 | .122 |  | 0.31 |
| Pain | 4235.00 | .285 |  | 0.23 |
| ***Burning*** | ***4619.00*** | ***.045*** |  | 0.42 |
| Warmth/Heat | 4233.00 | .491 |  | 0.22 |
| **Pinching** | **4688.50** | **.001** |  | 0.65 |
| Metallic/Iron taste | 4098.00 | .685 |  | 0.22 |
| Fatigue | 3855.00 | .377 |  | 0.22 |

# Aperiodic EEG (Δ Exponent and Offset) – Parallel mediation results for Dual Task

| **Supplementary Table 2.** Mediation model for aperiodic data in dual task. | | | | | | | |
| --- | --- | --- | --- | --- | --- | --- | --- |
|  | **b** | **SE** | **95% CI** | |  | **90% CI** | |
|  |  |  | **LLCI** | **ULCI** |  | **LLCI** | **ULCI** |
|  | *a-*paths: effect of Stimulation Condition on each mediator | | | | | | |
| a_1_: Δ Exponent (1-35 Hz) | -0.054 | 0.033 | -0.122 | 0.010 |  | -0.110 | 0.001 |
| a_2_: Δ Exponent (30-45 Hz) | -0.183 | 0.153 | -0.467 | 0.126 |  | -0.423 | 0.091 |
| a_3_: Δ Offset (1-35 Hz) | -0.008 | 0.045 | -0.097 | 0.082 |  | -0.083 | 0.067 |
| a_4_: Δ Offset (30-45 Hz) | -0.207 | 0.212 | -0.608 | 0.219 |  | -0.542 | 0.156 |
|  | *b-*paths: effect of each mediator on EV Slope | | | | | | |
| b1: Δ Exponent (1-35 Hz) | 0.066 | 0.156 | -0.231 | 0.377 |  | -0.169 | 0.338 |
| b2: Δ Exponent (30-45 Hz) | -0.052 | 0.144 | -0.349 | 0.211 |  | -0.309 | 0.172 |
| b3: Δ Offset (1-35 Hz) | -0.059 | 0.094 | -0.254 | 0.112 |  | -0.224 | 0.081 |
| b4: Δ Offset (30-45 Hz) | 0.033 | 0.097 | -0.147 | 0.231 |  | -0.116 | 0.203 |
|  | Total and indirect effects (Stimulation Condition > EV Slope) | | | | | | |
| c (Total Effect) | 0.003 | 0.008 | -0.012 | 0.019 |  | -0.010 | 0.016 |
| c' (Direct effect) | 0.004 | 0.008 | -0.013 | 0.020 |  | -0.010 | 0.017 |
|  | Indirect effects (Stimulation Condition > Mediator > EV Slope) | | | | | | |
| a_1_b_1_: tDCS 🡪 Δ Exponent (1-35 Hz) | -0.004 | 0.010 | -0.028 | 0.013 |  | -0.022 | 0.010 |
| a_2_b_2_: tDCS 🡪 Δ Exponent (30-45 Hz) | 0.009 | 0.034 | -0.062 | 0.091 |  | -0.045 | 0.068 |
| a_3_b_3_: tDCS 🡪 Δ Offset (1-35 Hz) | 0.001 | 0.005 | -0.010 | 0.012 |  | -0.006 | 0.010 |
| a_4_b_4_: tDCS 🡪 Δ Offset (30-45 Hz) | -0.007 | 0.029 | -0.075 | 0.055 |  | -0.056 | 0.041 |
| *Note. N* = 59. LLCI = lower limit of the CI, ULCI = upper limit of the CI. | | | | |  |  |  |

# Correlation between Aperiodic Parameters in the single and triple tasks

| **Supplementary Table 3.** Pearson correlation coefficients for Aperiodic Parameters in the single and triple task conditions. | | |
| --- | --- | --- |
|  | ***r*** | ***p*** |
|  |  |  |
| *Single task* | | |
| **Δ Aperiodic Exponent 1-35 – Δ Aperiodic Exponent 30-45** | **0.32** | **.015** |
| **Δ Aperiodic Exponent 1-35 – Δ Aperiodic Offset 1-35** | **0.84** | **< .001** |
| Δ Aperiodic Exponent 1-35 – Δ Aperiodic Offset 30-45 | 0.27 | .038 |
| Δ Aperiodic Exponent 30-45 – Δ Aperiodic Offset 1-35 | 0.18 | .166 |
| **Δ Aperiodic Exponent 30-45 – Δ Aperiodic Offset 30-45** | **0.98** | **< .001** |
| Δ Aperiodic Offset 1-35 – Δ Aperiodic Offset 30-45 | 0.25 | .061 |
| *Triple task* | | |
| **Δ Aperiodic Exponent 1-35 – Δ Aperiodic Exponent 30-45** | **0.33** | **.010** |
| **Δ Aperiodic Exponent 1-35 – Δ Aperiodic Offset 1-35** | **0.65** | **< .001** |
| **Δ Aperiodic Exponent 1-35 – Δ Aperiodic Offset 30-45** | **0.31** | **.020** |
| Δ Aperiodic Exponent 30-45 – Δ Aperiodic Offset 1-35 | -0.01 | .943 |
| **Δ Aperiodic Exponent 30-45 – Δ Aperiodic Offset 30-45** | **0.98** | **.001** |
| Δ Aperiodic Offset 1-35 – Δ Aperiodic Offset 30-45 | 0.13 | .348 |
| *Note****.*** Significant effects appear in bold. | | |

# Aperiodic EEG (Δ Exponent and Offset) – Single mediation results

## Triple Task:

| **Supplementary Table 4.** Single mediation model with Δ Aperiodic Exponent (1-35 Hz) in the triple task. | | | | | | | |
| --- | --- | --- | --- | --- | --- | --- | --- |
|  | **b** | **SE** | **90% CI** | |  | **95% CI** | |
|  |  |  | **LLCI** | **ULCI** |  | **LLCI** | **ULCI** |
| a: Stim. Cond > Δ AE 1-35 | -0.038 | 0.037 | -0.097 | 0.022 |  | -0.110 | 0.034 |
| b: Δ AE 1-35 > EV Slope | -0.005 | 0.036 | -0.068 | 0.051 |  | -0.081 | 0.061 |
| **c: Total Effect (Stim. Cond. > EV Slope)** | **0.018** | **0.010** | **0.002** | **0.035** |  | -0.002 | 0.038 |
| **c': Direct Effect (Stim. Cond. > EV Slope)** | **0.018** | **0.010** | **0.001** | **0.035** |  | -0.002 | 0.038 |
| ab: Stim. Cond. 🡪 Δ AE 1-35 > EV Slope | 0.000 | 0.002 | -0.003 | 0.004 |  | -0.003 | 0.005 |
| *Note. N* = 58. LLCI = lower limit of the CI, ULCI = upper limit of the CI. Significant effects appear in bold. | | | | | | | |

| **Supplementary Table 5.** Single mediation model with Δ Aperiodic Exponent (30-45 Hz) in the triple task. | | | | | | | |
| --- | --- | --- | --- | --- | --- | --- | --- |
|  | **b** | **SE** | **90% CI** | |  | **95% CI** | |
|  |  |  | **LLCI** | **ULCI** |  | **LLCI** | **ULCI** |
| **a: Stim. Cond > Δ AE 30-45** | **-0.313** | **0.171** | **-0.597** | **-0.033** |  | -0.639 | 0.015 |
| b: Δ AE 30-45 > EV Slope | -0.005 | 0.008 | -0.017 | 0.009 |  | -0.019 | 0.012 |
| **c: Total Effect (Stim. Cond. > EV Slope)** | **0.018** | **0.010** | **0.002** | **0.035** |  | -0.002 | 0.038 |
| **c': Direct Effect (Stim. Cond. > EV Slope)** | **0.017** | **0.010** | **0.000** | **0.034** |  | -0.004 | 0.037 |
| ab: Stim. Cond. 🡪 Δ AE 30-45 > EV Slope | 0.002 | 0.003 | -0.003 | 0.006 |  | -0.004 | 0.007 |
| *Note. N* = 58. LLCI = lower limit of the CI, ULCI = upper limit of the CI. Significant effects appear in bold. | | | | | | | |

| **Supplementary Table 6.** Single mediation model with Δ Aperiodic Offset (1-35 Hz) in the triple task. | | | | | | | |
| --- | --- | --- | --- | --- | --- | --- | --- |
|  | **b** | **SE** | **90% CI** | |  | **95% CI** | |
|  |  |  | **LLCI** | **ULCI** |  | **LLCI** | **ULCI** |
| **a: Stim. Cond > Δ AO 1-35** | **0.121** | **0.070** | **0.012** | **0.244** |  | -0.014 | 0.267 |
| b: Δ A:O 1-35 > EV Slope | -0.002 | 0.021 | -0.039 | 0.032 |  | -0.047 | 0.037 |
| **c: Total Effect (Stim. Cond. > EV Slope)** | **0.018** | **0.010** | **0.002** | **0.035** |  | -0.002 | 0.038 |
| **c': Direct Effect (Stim. Cond. > EV Slope)** | **0.018** | **0.010** | **0.001** | **0.035** |  | -0.002 | 0.039 |
| ab: Stim. Cond. 🡪 Δ AO 1-35 > EV Slope | 0.000 | 0.003 | -0.004 | 0.005 |  | -0.005 | 0.007 |
| *Note. N* = 58. LLCI = lower limit of the CI, ULCI = upper limit of the CI. Significant effects appear in bold. | | | | | | | |

| **Supplementary Table 7.** Single mediation model with Δ Aperiodic Offset (30-45 Hz) in the single task. | | | | | | | |
| --- | --- | --- | --- | --- | --- | --- | --- |
|  | **b** | **SE** | **90% CI** | |  | **95% CI** | |
|  |  |  | **LLCI** | **ULCI** |  | **LLCI** | **ULCI** |
| a: Stim. Cond > Δ AO 30-45 | -0.302 | 0.250 | -0.729 | 0.091 |  | -0.783 | 0.195 |
| b: Δ AO 30-45 > EV Slope | -0.003 | 0.005 | -0.011 | 0.007 |  | -0.013 | 0.008 |
| **c: Total Effect (Stim. Cond. > EV Slope)** | **0.018** | **0.010** | **0.002** | **0.035** |  | -0.002 | 0.038 |
| **c': Direct Effect (Stim. Cond. > EV Slope)** | **0.017** | **0.010** | **0.001** | **0.034** |  | -0.003 | 0.037 |
| ab: Stim. Cond. 🡪 Δ AO 30-45 > EV Slope | 0.001 | 0.002 | -0.002 | 0.004 |  | -0.003 | 0.005 |
| *Note. N* = 58. LLCI = lower limit of the CI, ULCI = upper limit of the CI. Significant effects appear in bold. | | | | | | | |

## Single Task:

| **Supplementary Table 8.** Single mediation model with Δ Aperiodic Exponent (1-35 Hz) in the single task. | | | | | | | |
| --- | --- | --- | --- | --- | --- | --- | --- |
|  | **b** | **SE** | **90% CI** | |  | **95% CI** | |
|  |  |  | **LLCI** | **ULCI** |  | **LLCI** | **ULCI** |
| a: Stim. Cond > Δ AE 1-35 | 0.019 | 0.049 | -0.063 | 0.096 |  | -0.080 | 0.117 |
| b: Δ AE 1-35 > EV Slope | -0.021 | 0.021 | -0.060 | 0.009 |  | -0.067 | 0.013 |
| **c: Total Effect (Stim. Cond. > EV Slope)** | -0.010 | 0.008 | -0.023 | 0.003 |  | -0.025 | 0.006 |
| **c': Direct Effect (Stim. Cond. > EV Slope)** | -0.010 | 0.008 | -0.023 | 0.004 |  | -0.025 | 0.006 |
| ab: Stim. Cond. 🡪 Δ AE 1-35 > EV Slope | 0.000 | 0.002 | -0.004 | 0.001 |  | -0.005 | 0.002 |
| *Note. N* = 58. LLCI = lower limit of the CI, ULCI = upper limit of the CI. Significant effects appear in bold. | | | | | | | |

| **Supplementary Table 9.** Single mediation model with Δ Aperiodic Exponent (30-45 Hz) in the single task. | | | | | | | |
| --- | --- | --- | --- | --- | --- | --- | --- |
|  | **b** | **SE** | **90% CI** | |  | **95% CI** | |
|  |  |  | **LLCI** | **ULCI** |  | **LLCI** | **ULCI** |
| **a: Stim. Cond > Δ AE 30-45** | **-0.244** | **0.135** | **-0.454** | **-0.014** |  | -0.508 | 0.010 |
| b: Δ AE 30-45 > EV Slope | 0.000 | 0.007 | -0.013 | 0.011 |  | -0.015 | 0.014 |
| c: Total Effect (Stim. Cond. > EV Slope) | -0.010 | 0.008 | -0.023 | 0.003 |  | -0.025 | 0.006 |
| c': Direct Effect (Stim. Cond. > EV Slope) | -0.010 | 0.008 | -0.023 | 0.004 |  | -0.026 | 0.006 |
| ab: Stim. Cond. 🡪 Δ AE 30-45 > EV Slope | 0.000 | 0.002 | -0.004 | 0.003 |  | -0.005 | 0.004 |
| *Note. N* = 58. LLCI = lower limit of the CI, ULCI = upper limit of the CI. Significant effects appear in bold. | | | | | | | |

| **Supplementary Table 10.** Single mediation model with Δ Aperiodic Offset (1-35 Hz) in the single task. | | | | | | | |
| --- | --- | --- | --- | --- | --- | --- | --- |
|  | **b** | **SE** | **90% CI** | |  | **95% CI** | |
|  |  |  | **LLCI** | **ULCI** |  | **LLCI** | **ULCI** |
| a: Stim. Cond > Δ AO 1-35 | .027 | .073 | -.094 | 0.150 |  | -.113 | .165 |
| b: Δ AO 1-35 > EV Slope | -.009 | .013 | -.030 | 0.012 |  | -.034 | .018 |
| c: Total Effect (Stim. Cond. > EV Slope) | -.010 | .008 | -.023 | 0.003 |  | -.025 | .006 |
| c': Direct Effect (Stim. Cond. > EV Slope) | -.010 | .008 | -.023 | 0.004 |  | -.025 | .006 |
| ab: Stim. Cond. 🡪 Δ AO 1-35 > EV Slope | .000 | .001 | -.003 | 0.001 |  | -.004 | .002 |
| *Note. N* = 58. LLCI = lower limit of the CI, ULCI = upper limit of the CI. | | | | | | | |

| **Supplementary Table 11.** Single mediation model with Δ Aperiodic Offset (30-45 Hz) in the single task. | | | | | | | |
| --- | --- | --- | --- | --- | --- | --- | --- |
|  | **b** | **SE** | **90% CI** | |  | **95% CI** | |
|  |  |  | **LLCI** | **ULCI** |  | **LLCI** | **ULCI** |
| **a: Stim. Cond > Δ AO 30-45** | **-0.364** | **0.196** | **-0.676** | **-0.041** |  | -0.742 | 0.006 |
| b: Δ AO 30-45 > EV Slope | 0.000 | 0.005 | -0.009 | 0.008 |  | -0.011 | 0.010 |
| c: Total Effect (Stim. Cond. > EV Slope) | -0.010 | 0.008 | -0.023 | 0.003 |  | -0.025 | 0.006 |
| c': Direct Effect (Stim. Cond. > EV Slope) | -0.010 | 0.008 | -0.023 | 0.004 |  | -0.026 | 0.006 |
| ab: Stim. Cond. 🡪 Δ AO 30-45 > EV Slope | 0.000 | 0.002 | -0.004 | 0.004 |  | -0.005 | 0.004 |
| *Note. N* = 58. LLCI = lower limit of the CI, ULCI = upper limit of the CI. Significant effects appear in bold. | | | | | | | |

# Periodic EEG (Δ Alpha Power) – Mediation results

A parallel mediation model including both Δ Alpha Power and Δ Gamma Power could not be run as too few observations remained in Δ Gamma Power. The mediation model with Δ Alpha Power as a mediator in the triple task (see **Supplementary Fig. 2.A**), revealed a significant negative effect of the Stimulation Condition on Δ Alpha Power. As shown in **Supplementary Fig. 2.B**, the increment of alpha power was significantly reduced in the group receiving anodal HD-tDCS compared to the sham group in the triple task condition, thus replicating previous results exploring alpha power without parametrizing the power spectra (Hemmerich et al., 2023; Luna et al., 2020). The direct effect of Stimulation Condition on EV Slope remained significant (see **Supplementary Table 12**), however, neither the b-path (effect of Δ Alpha Power on EV Slope) nor a mediated effect (indirect effect of Stimulation Condition on EV Slope via the Δ Alpha Power) was observed. Similarly, in the single and dual tasks alpha power also increased from the pre- to the post-stimulation recording. However as can be seen in **Supplementary Fig. 2.B**, this was not affected by Stimulation Condition (i.e., the *a*-paths were non-significant). Furthermore, for the single and dual tasks, the b-path, direct or indirect effects were also not significant, when introducing Δ Alpha Power as a mediator in the relationship between Stimulation Condition and EV Slope. Full results are reported below in **Supplementary Tables 12-14.**


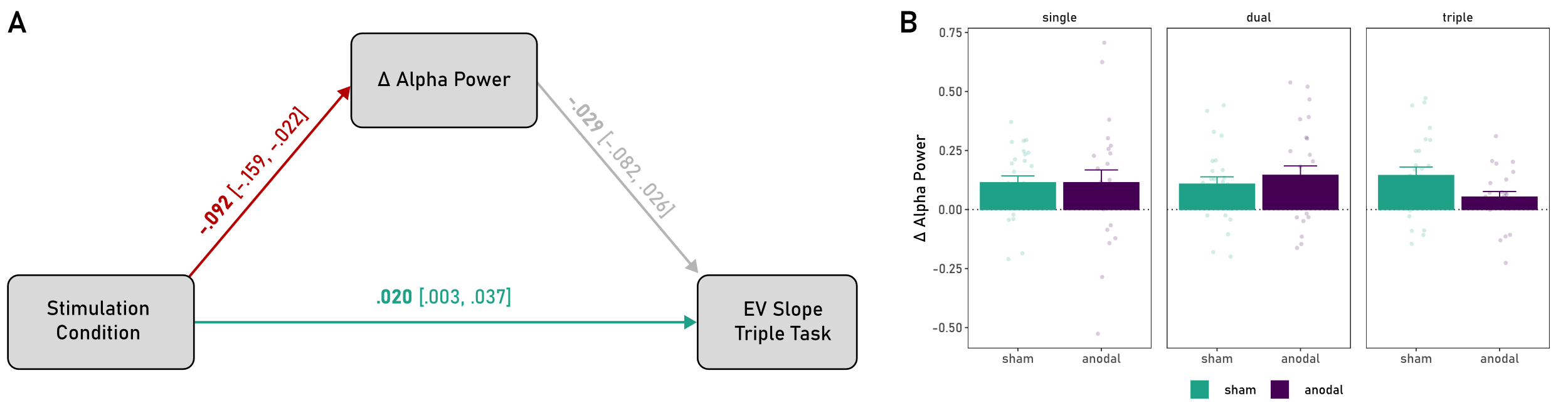


**Fig. 2. (A)** Mediation results for periodic EEG data (alpha power) in the triple task condition. **(B)** A significant *a*-path was observed in the triple task, as the alpha power increment from pre- to post-stimulation was significantly decreased in the triple task condition.

| **Supplementary Table 12.** Mediation model for periodic data in the triple task. | | | | | | | |
| --- | --- | --- | --- | --- | --- | --- | --- |
|  | **b** | **SE** | **90% CI** | |  | **95% CI** | |
|  |  |  | **LLCI** | **ULCI** |  | **LLCI** | **ULCI** |
| **a: Stim. Cond. > Δ Alpha Power** | **-0.092** | **0.043** | **-0.159** | **-0.022** |  | **-0.175** | **-0.012** |
| b: Δ Alpha Power > EV Slope | -0.029 | 0.032 | -0.082 | 0.026 |  | -0.094 | 0.037 |
| **c: Total Effect (Stim. Cond. > EV Slope)** | **0.023** | **0.010** | **0.006** | **0.039** |  | **0.003** | **0.042** |
| **c': Direct effect (Stim. Cond. > EV Slope)** | **0.020** | **0.010** | **0.003** | **0.037** |  | -0.001 | 0.040 |
| ab: Stim. Cond. 🡪 Δ Alpha Power > EV Slope | 0.003 | 0.003 | -0.002 | 0.009 |  | -0.004 | 0.010 |
| *Note. N* = 50. LLCI = lower limit of the CI, ULCI = upper limit of the CI. Significant effects appear in bold. | | | | | | | |

| **Supplementary Table 13.** Mediation model for periodic data in the single task. | | | | |
| --- | --- | --- | --- | --- |
|  | **b** | **SE** | **95% CI** | |
|  |  |  | **LLCI** | **ULCI** |
| a: Stim. Cond > Δ Alpha Power | 0.011 | 0.013 | -0.105 | 0.128 |
| b: Δ Alpha Power > EV Slope | 0.025 | 0.026 | -0.013 | 0.068 |
| c: Total Effect (Stim. Cond. > EV Slope) | -0.011 | 0.009 | -0.029 | 0.006 |
| c': Direct effect (Stim. Cond. > EV Slope) | -0.012 | 0.009 | -0.029 | 0.006 |
| ab: Stim. Cond. 🡪 Δ Alpha Power > EV Slope | 0.000 | 0.002 | -0.004 | 0.004 |
| *Note. N* = 49. LLCI = lower limit of the CI, ULCI = upper limit of the CI.. | | | | |

| **Supplementary Table 14.** Mediation model for periodic data in the dual task. | | | | |
| --- | --- | --- | --- | --- |
|  | **b** | **SE** | **95% CI** | |
|  |  |  | **LLCI** | **ULCI** |
| a: Stim. Cond > Δ Alpha Power | 0.038 | 0.039 | -0.054 | 0.132 |
| b: Δ Alpha Power > EV Slope | 0.022 | 0.023 | -0.018 | 0.061 |
| c: Total Effect (Stim. Cond. > EV Slope) | 0.006 | 0.009 | -0.011 | 0.023 |
| c': Direct effect (Stim. Cond. > EV Slope) | 0.005 | 0.009 | -0.012 | 0.023 |
| ab: Stim. Cond. 🡪 Δ Alpha Power > EV Slope | 0.001 | 0.002 | -0.002 | 0.005 |
| *Note. N* = 52. LLCI = lower limit of the CI, ULCI = upper limit of the CI. | | | | |

# Aperiodic EEG (Baseline Exponent and Offset) - Moderation results

Models including the direct effect of Stimulation Condition on EV, and both Task Type and baseline values of either aperiodic index (exponent and offset from the 1-35 and 30-45 Hz range), revealed no significant moderated moderation (i.e., interaction of Stimulation Condition, Task Type, and baseline; see full results in **Supplementary Tables 14-17**). Thus, the effect of tDCS on the EV decrement does not seem to be moderated by baseline EEG aperiodic data.

| **Supplementary Table 15.** Moderation model for Baseline Aperiodic Exponent (1-35 Hz) | | | | | | | |
| --- | --- | --- | --- | --- | --- | --- | --- |
|  | **b** | **SE** | **90% CI** | |  | **95% CI** | |
|  |  |  | **LLCI** | **ULCI** |  | **LLCI** | **ULCI** |
| *Outcome variable: EV Slope* | | | | | | | |
| Stimulation Condition | -0.007 | 0.008 | -0.021 | 0.006 |  | -0.023 | 0.008 |
| Baseline Exponent | 0.039 | 0.039 | -0.035 | 0.093 |  | -0.044 | 0.109 |
| Stimulation Condition × Baseline Exponent | -0.012 | 0.056 | -0.087 | 0.098 |  | -0.103 | 0.118 |
| Dual Task | -0.002 | 0.007 | -0.014 | 0.009 |  | -0.017 | 0.012 |
| **Triple Task** | **-0.020** | **0.009** | **-0.035** | **-0.006** |  | **-0.038** | **-0.002** |
| Stimulation Condition × Dual Task | 0.011 | 0.011 | -0.009 | 0.028 |  | -0.013 | 0.032 |
| **Stimulation Condition × Triple Task** | **0.025** | **0.013** | **0.004** | **0.047** |  | **0.000** | **0.051** |
| Baseline Exponent × Dual Task | -0.050 | 0.061 | -0.163 | 0.037 |  | -0.192 | 0.055 |
| Baseline Exponent × Triple Task | -0.077 | 0.051 | -0.151 | 0.014 |  | -0.165 | 0.030 |
| Stimulation Condition × Baseline Exponent × Dual Task | -0.002 | 0.080 | -0.132 | 0.133 |  | -0.159 | 0.160 |
| Stimulation Condition × Baseline Exponent × Triple Task | -0.004 | 0.081 | -0.153 | 0.117 |  | -0.180 | 0.138 |
| *Note. N* = 175. LLCI = lower limit of the CI, ULCI = upper limit of the CI. | | | | | | | |

| **Supplementary Table 16.** Moderation model for Baseline Aperiodic Exponent (30-45 Hz) | | | | | | | |
| --- | --- | --- | --- | --- | --- | --- | --- |
|  | **b** | **SE** | **90% CI** | |  | **95% CI** | |
|  |  |  | **LLCI** | **ULCI** |  | **LLCI** | **ULCI** |
| *Outcome variable: EV Slope* | | | | | | | |
| Stimulation Condition | -0.011 | 0.008 | -0.023 | 0.003 |  | -0.026 | 0.006 |
| **Baseline Exponent** | **0.011** | **0.005** | **0.000** | **0.017** |  | -0.003 | 0.020 |
| Stimulation Condition × Baseline Exponent | -0.004 | 0.010 | -0.019 | 0.013 |  | -0.023 | 0.016 |
| Dual Task | -0.005 | 0.006 | -0.015 | 0.006 |  | -0.018 | 0.008 |
| **Triple Task** | **-0.022** | **0.008** | **-0.035** | **-0.007** |  | **-0.037** | **-0.003** |
| Stimulation Condition × Dual Task | 0.013 | 0.011 | -0.007 | 0.030 |  | -0.010 | 0.035 |
| **Stimulation Condition × Triple Task** | **0.028** | **0.013** | **0.007** | **0.049** |  | 0.004 | 0.053 |
| **Baseline Exponent × Dual Task** | **-0.023** | **0.011** | **-0.041** | **-0.006** |  | **-0.046** | **-0.002** |
| **Baseline Exponent × Triple Task** | **-0.023** | **0.011** | **-0.040** | **-0.005** |  | **-0.046** | **-0.001** |
| Stimulation Condition × Baseline Exponent × Dual Task | 0.021 | 0.014 | -0.001 | 0.046 |  | -0.008 | 0.051 |
| Stimulation Condition × Baseline Exponent × Triple Task | 0.011 | 0.017 | -0.018 | 0.036 |  | -0.024 | 0.042 |
| *Note. N* = 175. LLCI = lower limit of the CI, ULCI = upper limit of the CI. | | | | | | | |

| **Supplementary Table 17.** Moderation model for Baseline Aperiodic Offset (1-35 Hz) | | | | |
| --- | --- | --- | --- | --- |
|  | **b** | **SE** | **95% CI** | |
|  |  |  | **LLCI** | **ULCI** |
| *Outcome variable: EV Slope* | | | | |
| Stimulation Condition | -0.008 | 0.008 | -0.024 | 0.008 |
| Baseline Offset | 0.015 | 0.020 | -0.017 | 0.069 |
| Stimulation Condition × Baseline Offset | 0.004 | 0.034 | -0.059 | 0.084 |
| Dual Task | -0.004 | 0.007 | -0.019 | 0.009 |
| **Triple Task** | **-0.020** | **0.009** | **-0.037** | **-0.002** |
| Stimulation Condition × Dual Task | 0.013 | 0.011 | -0.012 | 0.034 |
| **Stimulation Condition × Triple Task** | **0.025** | **0.013** | **0.001** | **0.050** |
| Baseline Offset × Dual Task | 0.017 | 0.030 | -0.055 | 0.069 |
| Baseline Offset × Triple Task | -0.038 | 0.034 | -0.108 | 0.028 |
| Stimulation Condition × Baseline Offset × Dual Task | -0.051 | 0.047 | -0.147 | 0.038 |
| Stimulation Condition × Baseline Offset × Triple Task | -0.020 | 0.052 | -0.131 | 0.077 |
| *Note. N* = 175. LLCI = lower limit of the CI, ULCI = upper limit of the CI. | | | | |

| **Supplementary Table 18.** Moderation model for Baseline Aperiodic Offset (30-45 Hz) | | | | |
| --- | --- | --- | --- | --- |
|  | **b** | **SE** | **95% CI** | |
|  |  |  | **LLCI** | **ULCI** |
| *Outcome variable: EV Slope* | | | | |
| Stimulation Condition | -0.011 | 0.008 | -0.027 | 0.006 |
| Baseline Offset | 0.007 | 0.004 | -0.003 | 0.012 |
| Stimulation Condition × Baseline Offset | -0.002 | 0.006 | -0.014 | 0.011 |
| Dual Task | -0.005 | 0.007 | -0.018 | 0.009 |
| **Triple Task** | **-0.022** | **0.009** | **-0.037** | **-0.002** |
| Stimulation Condition × Dual Task | 0.013 | 0.011 | -0.010 | 0.036 |
| **Stimulation Condition × Triple Task** | **0.028** | **0.013** | **0.003** | **0.053** |
| Baseline Offset × Dual Task | -0.014 | 0.007 | -0.028 | 0.001 |
| Baseline Offset × Triple Task | -0.013 | 0.007 | -0.027 | 0.001 |
| Stimulation Condition × Baseline Offset × Dual Task | 0.013 | 0.009 | -0.005 | 0.031 |
| Stimulation Condition × Baseline Offset × Triple Task | 0.005 | 0.011 | -0.017 | 0.026 |
| *Note. N* = 175. LLCI = lower limit of the CI, ULCI = upper limit of the CI. | | | | |

# Periodic EEG (Baseline Alpha Power) – Moderation results

Aperiodic baseline data did also not show a significantly moderated moderation (i.e., interaction of Stimulation Condition, Task Type, and Baseline Alpha Power; see **Supplementary Table 18**). The results suggest that the effect of tDCS on the EV decrement does not seem to be moderated by baseline EEG periodic data (using alpha power as a representative of periodic data).

| **Supplementary Table 19.** Moderation model for periodic data for Baseline Alpha Power | | | | |
| --- | --- | --- | --- | --- |
|  | **b** | **SE** | **95% CI** | |
|  |  |  | **LLCI** | **ULCI** |
| *Outcome variable: EV Slope* | | | | |
| Stimulation Condition | -0.008 | 0.009 | -0.026 | 0.007 |
| Baseline Alpha Power | 0.019 | 0.020 | -0.030 | 0.049 |
| Stimulation Condition × Baseline Alpha Power | -0.033 | 0.031 | -0.092 | 0.034 |
| Dual Task | -0.006 | 0.007 | -0.021 | 0.006 |
| Triple Task | -0.016 | 0.009 | -0.034 | 0.000 |
| Stimulation Condition × Dual Task | 0.015 | 0.012 | -0.007 | 0.040 |
| Stimulation Condition × Triple Task | 0.026 | 0.014 | -0.001 | 0.055 |
| Baseline Alpha Power × Dual Task | 0.035 | 0.024 | -0.005 | 0.090 |
| Baseline Alpha Power × Triple Task | -0.029 | 0.042 | -0.113 | 0.046 |
| Stimulation Condition × Baseline Alpha Power × Dual Task | -0.028 | 0.041 | -0.114 | 0.055 |
| Stimulation Condition × Baseline Alpha Power × Triple Task | 0.053 | 0.058 | -0.065 | 0.171 |
| *Note. N* = 161. LLCI = lower limit of the CI, ULCI = upper limit of the CI. | | | | |
